# Tubulin and GTP Are Crucial Elements for Postsynaptic Density Construction and Aggregation

**DOI:** 10.1111/jnc.70085

**Published:** 2025-05-21

**Authors:** Tatsuo Suzuki, Toshihiro Fujii, Kiyokazu Kametani, Weidong Li, Katsuhiko Tabuchi

**Affiliations:** ^1^ Department of Molecular and Cellular Physiology Shinshu University School of Medicine Matsumoto Japan; ^2^ Shinshu University, Textile Science and Technology Ueda Japan; ^3^ Department of Veterinary Anatomy, Faculty of Veterinary Medicine Rakuno Gakuen University 582 Midori Cho Ebetsu Hokkaido Japan; ^4^ Center for Brain Health and Brain Technology, Global Institute of Future Technology, Institute of Psychology and Behavioral Science Shanghai Jiao Tong University Shanghai China; ^5^ Institute for Biomedical Sciences Interdisciplinary Cluster for Cutting Edge Research Shinshu University Matsumoto Japan; ^6^ Department of Biological Sciences for Intractable Neurological Diseases, Institute for Biomedical Sciences Interdisciplinary Cluster for Cutting Edge Research Shinshu University Matsumoto Japan; ^7^ Department of Molecular and Cellular Physiology, Shinshu University Academic Assembly, Institute of Medicine Shinshu University Academic Assembly Matsumoto Japan

**Keywords:** microtubule, postsynaptic density, PSD aggregation, PSD lattice, tubulin

## Abstract

In our previous experiments on the postsynaptic density lattice (PSDL), which is thought to serve as the backbone structure for the PSD, we suggested that tubulin plays a fundamental role in the PSD structure at excitatory synapses. In this study, we further reveal an unrecognized characteristic of tubulin within the PSD. First, using electron microscopy, we identified an interaction between postsynaptic structures (PSDL and PSD) and polymerizing microtubules, which led to the binding of polymerizing microtubules to PSDL and PSD. In turn, this interaction induced changes in the microtubule morphology. These results support earlier findings suggesting that microtubules transiently intruding into the spine head can associate with PSDs, inducing structural changes in the PSD. Next, we observed that the structural integrity of both PSD and PSDL was compromised upon exposure to GTP and microtubule‐affecting reagents. These findings reinforce the idea that tubulin is a crucial building block of the PSD architecture. Moreover, we found that PSD aggregation was enhanced following interactions with polymerizing tubulin and was disintegrated upon treatment with GTP and microtubule‐affecting reagents. These results indicate that microtubules also play a key role in PSD aggregation in vitro. Collectively, our study highlights the involvement of tubulin in the construction, function (specifically its interaction with polymerizing microtubules), and aggregation of the PSD, which may impact both physiological and pathological conditions. Furthermore, our in vitro findings suggest that GTP can either destroy or induce the enlargement and reorganization of PSD structures, depending on its interaction with growing microtubules.

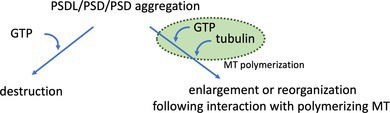

## Graphical abstract

As illustrated in the graphical abstract, postsynaptic structures were incubated with GTP either in the presence or absence of polymerizing tubulin. These conditions led to distinct outcomes: structural destruction in the absence of tubulin, and enlargement or reorganization in its presence. These findings suggest that the postsynaptic density (PSD) is inherently dynamic, constantly balancing between disassembly and reconstruction. This balance appears to be modulated by the presence of polymerizing microtubules (MTs) and GTP. We propose a model in which GTP plays a central role in the maintenance and remodeling of PSD normal structure and aggregation.

**Abbreviations**
GTB: general tubulin buffer
IAA: iodoacetamide
MAP: microtubule‐associated protein
MT: microtubule
NGS: normal goat serum
OG: n‐octyl‐β‐D‐glucoside
OG12: fraction 12 prepared from SPM after OG treatment (one type of PSD preparation)
PSD: postsynaptic density
PSDL: PSD lattice
SPM: synaptic plasma membrane
TX‐PSD: PSD prepared by Triton X‐100 treatment

## 1 Introduction

A complete understanding of the structure of the postsynaptic density (PSD) is indispensable to elucidate the molecular mechanisms underlying changes in the spine and PSD during the expression of synaptic plasticity. The molecular mechanisms underlying actin dynamics in spine changes have been well‐established (Bosch et al. 2014; Sekino et al. 2007; Spence and Soderling 2015); however, the mechanisms underlying changes in PSD structure have not been clearly elucidated until recently.

The presence of tubulin in and around the PSD has long been a point of debate, despite the identification of tubulin immunoreactivity in the PSD as early as 1975 (Suzuki et al. 2021). In 2008, tubulin present in the PSD was brought to the limelight again due to the transient appearance of MTs in dendritic spines during synaptic plasticity expression (Gu et al. 2008; Hu et al. 2008; Jaworski et al. 2009). More recently, the localization of tau, a microtubule‐associated protein (MAP), to the postsynaptic region has become apparent (Frandemiche et al. 2014; Ittner and Ittner 2018; Ittner et al. 2010), sparking a growing interest in the roles of both tau and tubulin at this site. Furthermore, there is increasing recognition of the importance of microtubule (MT) modulation in the regulation of various brain functions and dysfunctions, particularly in relation to synapses and dendritic spines (Pena‐Ortega et al. 2022; Verstraelen et al. 2017). Tubulin has been demonstrated to be present in the PSD in vivo through immuno‐gold labeling (Suzuki et al. 2021), and its presence in purified PSD preparations does not appear to be merely due to contamination (Ratner and Mahler 1983; Somerville et al. 1984). The accumulated body of research on tubulin now provides a solid basis for renewed investigation into the role of tubulin in the PSD.

We recently proposed a model for PSD architecture based on the purification and characterization of the PSD lattice (PSDL), a backbone structure for the PSD in brain synapses (Suzuki et al. 2021). We proposed a model in which tubulin‐based PSDL structures may play important roles as platforms to which PSD scaffold/adaptor proteins and various PSD‐functioning molecules become associated while synapses mature and reorganize (it is referred to here as the PSDL platform model). We also referred to the possibility that tubulin in its non‐MT form is a major core in the fundamental PSDL skeleton, which we defined as an essential skeletal structure for PSDs.

In this article, we report further information on tubulin in the PSD and PSDL, that is, (1) the interaction of the PSD and PSDL with polymerizing MTs, (2) GTP‐ and MT‐affective reagent‐induced modifications in PSD/PSDL structure and integrity, and (3) the involvement of tubulin in the formation of PSD aggregates and their destruction in the presence of GTP and MT‐affective reagents. These findings may provide new insights into the role of tubulin in the postsynaptic region.

## 2 Materials and Methods

### 2.1 Materials

The chemicals and antibodies used in this study are listed in Tables 1 and 2. All the chemicals listed in Table 1 are of reagent grade.

**TABLE 1:** List of major chemicals used in this study.

Chemicals (abbreviated names)	Code no.	Providers
ATP	A‐2383	Shigma Chemical Company (St. Louis, Mo, USA)
DMSO	045‐07215	WAKO Pure Chemical Industries. Ltd. (Osaka, Japan)
EM grid (F‐400)	OF29T	NISSHIN EM Co. Ltd. (Tokyo, Japan)
Glutaraldehyde	077‐01933	WAKO Pure Chemical Industries. Ltd. (Osaka, Japan)
Glycerol	17 018‐25	Nakalai Tesque Inc. (Kyoto, Japan)
GTP solution	NU‐1012	Jena Bioscience GmbH (Jena, Germany)
GDP	ab146529	Abcam (Cambridge, England)
Guanosine 5′‐[β,γ‐imido]triphosphate trisodium salt (GMP‐PNP)	G0635	Sigma‐Aldrich (St Louis, MO, USA)
Iodoacetamide (IAA)	095‐02891	WAKO Pure Chemical Industries. Ltd. (Osaka, Japan)
Nano‐W	2018	Molecular probes (Yaphank, NY, USA)
Nocodazole	M1404	Sigma‐Aldrich (St. Louis, MO, USA)
Normal goat serum	0929391‐CF/5006‐1380	MP Biomedicals (cappel) (Irvine, CA, USA)
n‐Octyl‐β‐D‐glucoside (OG)	346‐05033	Dojindo Laboratories (Kumamoto, Japan)
O,O′‐Bis(2‐aminoethyl)ethylene‐glycol‐N,N,N′,N′‐tetraacetic acid (EGTA)	346‐01312	Dojindo Laboratories (Kamimashiki‐gun, Japan)
Oriole	161‐0495	Bio Rad (Hercules, CA, USA)
Paclitaxel	10 461	Cayman chemical (Ann Arbor, MI, USA)
PIPES	347‐02224	Dojindo Laboratories (Kamimashiki‐gun, Japan)
Polystyrene balance dish	AS‐DS	BIO‐BIK Co (Nagano, Japan)
Polystyene latex	1252	NISSHIN EM Co. Ltd. (Tokyo, Japan)
Protease inhibitor cocktail	P8340	Sigma‐Aldrich (St Louis, MO, USA)
TritonX‐100 (TX‐100)	581‐81 705	WAKO Pure Chemical Industries. Ltd. (Osaka, Japan)
Tubulin (> 99% pure)	T240	Cytoskeleton Inc. (Denver, CO, USA)
Tubulin MAP‐rich	ML116	Cytoskeleton Inc. (Denver, CO, USA)

**TABLE 2:** List of antibodies used for immuno‐gold electron microscopy.

Antibodies used for immuno‐gold labeling	RRID (−) not found or no exact match	Catalog or clone No.	Company	Mono or poly	Animals for antibody production	Dilution used
Anti‐tubulin*	Not registered	(1)	Produced by Dr. Fujii, Shinshu University	Polyclonal	Rabbit	1/20
Anti‐β‐tubulin	AB_609915	T‐5201	Sigma (St. Louis, MO)	Monoclonal	Mouse	1/20
Anti‐mouse IgG (H + L)‐gold label	(−)	EMGMHL10	BBI solutions (Cardiff, UK)	Polyclonal	Goat	1/50
Anti‐rabbit IgG (H + L)‐gold label	(−)	EMGAR10	BBI solutions (Cardiff, UK)	Polyclonal	Goat	1/50

*Note:* (1) Anti‐tubulin antibody was produced in rabbit using pig tubulin as antigen and affinity‐purified (Liu et al. 2013).

Abbreviation: HRPO, horseradish peroxidase.

### 2.2 Ethical Approval/Animals

Animals were handled in accordance with the guidelines of the Regulations for Animal Experimentation of Shinshu University. The animal handling protocol was approved by the Committee for Animal Experiments of Shinshu University (approval no. 240066). Based on national regulations and guidelines, all experimental procedures were reviewed by the Committee for Animal Experiments and finally approved by the president of Shinshu University.

Wistar rats (male, 6 weeks old, body weight: 150 ± 8 g, specific pathogen‐free) were purchased from Japan SLC Inc. (Hamamatsu, Japan). The brains of 6‐week‐old rats were collected by decapitation without using any anesthetics on the day of delivery.

### 2.3 Preparation of Synaptic Plasma Membranes and Conventional PSDs

Synaptic plasma membranes (SPMs) were prepared from 10 Wistar rats in the presence of the antioxidant reagent, iodoacetamide (IAA), as previously described (Suzuki et al. 2018; Suzuki et al. 2019). Purified SPMs were stored unfrozen in buffers containing 50% glycerol at −80°C.

“Conventional” TX‐PSD was prepared from the forebrains of nineteen 6‐week‐old rats through the treatment of synaptosomes with 0.5% Triton X‐100 (Cohen et al. 1977; Suzuki et al. 2019). “Conventional” n‐octyl‐β‐D‐glucoside‐insoluble PSD (OG‐PSD) was prepared from the forebrains of five 6‐week‐old rats following the procedure for “conventional” TX‐PSD purification, using OG (1%) instead of Triton X‐100 (0.5%) (Suzuki et al. 2018; Suzuki et al. 2019; Suzuki et al. 2021). Conventional PSDs were prepared in the absence of IAA following the original protocol (Cohen et al. 1977), supplemented with 50% glycerol, and stored at −80°C.

### 2.4 Purification of PSDLs and OG12, a Type of OG‐Insoluble PSD Preparation

PSDL preparations (OG‐11U‐IS) were purified from SPMs (see the above section) of Wistar rats following our published protocol in the presence of IAA (Suzuki et al. 2019). OG12, another type of PSD preparation, is purified from SPM (see the above section) of Wistar rats and is a pellet obtained after sucrose density gradient ultracentrifugation during PSDL purification. Of note, OG‐PSD (see the above section) and OG12 differ in their purification protocols. PSDLs and OG12 were finally suspended in 5 mM HEPES/KOH (pH 7.4) containing 50% glycerol and stored at −30°C. The morphologies of PSDLs and OG12 did not change for at least 1 year after purification.

### 2.5 Evaluation of the Interaction Between PSDLs or PSDs and Polymerizing MTs

Lyophilized tubulin (T240 or ML116; Cytoskeleton Inc., Denver, CO, USA) was reconstituted according to the manufacturer's instructions in general tubulin buffer (GTB) (80 mM PIPES, pH 6.9; 2 mM MgCl_2_; 0.5 mM EGTA) supplemented with 1 mM GTP to final concentrations of 10 mg/mL (T240) or 5 mg/mL (ML116).

The reconstituted tubulin solution was rapidly frozen in liquid nitrogen or dry ice and stored at −80°C. On the day of the experiment, the defrosted tubulin solution was mixed with glycerol and GTP at 0°C (final solution: GTB containing 1 mM GTP, 5% glycerol and 5 mg/mL, or 1 or 2 mg/mL of pure tubulin (T240) or MAP‐rich tubulin (ML116), respectively). Tubulin polymerization was performed either in a small plastic tube or in a droplet formed on a polystyrene plastic balance dish.

**FIGURE 1:**
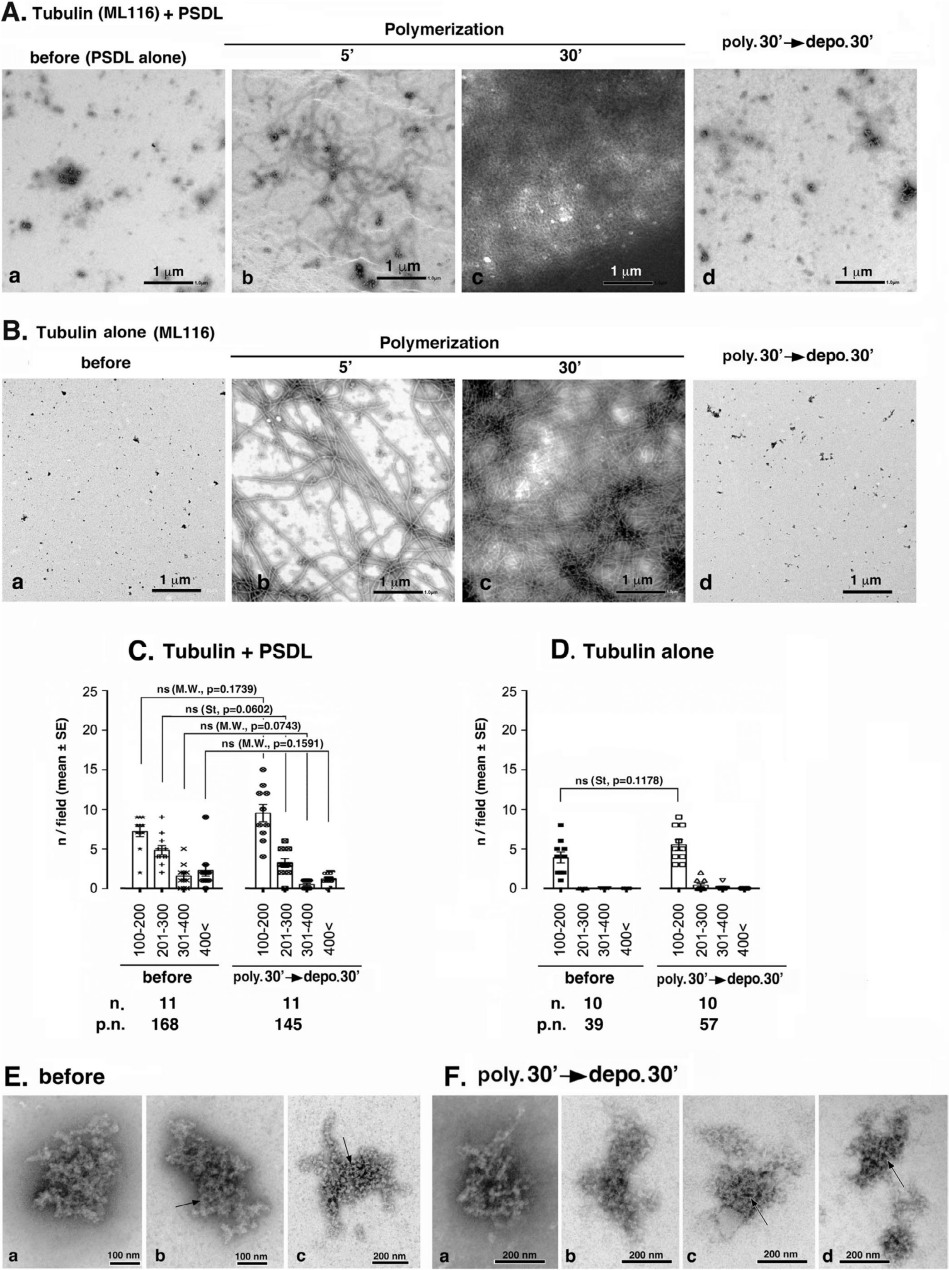
Low‐magnification images for the analyses of the interaction between PSDLs and MTs. (A, B) Time course of MT polymerization in the presence or absence of PSDLs. Tubulin polymerization (ML116, 2 mg/mL) was performed in the presence (A) or absence (B) of PSDLs. The PSDL‐laden membrane was blocked at 0°C and 37°C before contacting with tubulin‐containing droplets (see Figure S1B for the effect of blocking the Formvar membrane). PSDL‐laden grids were contacted with tubulin‐containing droplets at 0°C and then transferred to a pre‐heated aluminum block at 37°C to initiate tubulin polymerization. The PSDL‐unladen grid membrane was not blocked and brought into contact with tubulin‐containing droplets for 30 s at specific time points. “Before” samples in the “Tubulin+PSDL” experiment were not put in contact with tubulin‐containing droplets. Samples on the grids were fixed for 5 min and negatively stained with nano‐W. (C, D) Quantification of changes in particle size. Experiments in the presence (C) or absence (D) of PSDLs were performed as stated above. EM images of the negatively stained specimens were captured randomly at an 8000× magnification. Particles of different sizes (> 100 nm) were counted manually, as it was not possible to simultaneously measure structures of varying sizes within the same visual field under identical contrast conditions using ImageJ for particle analysis. The vertical line represents the number of particles in a field imaged at 8000× magnification. The number of 8000× magnified images (n) and the total number of particles (p.n.) are indicated at the bottom. Significance was tested using Student's *t* test or the Mann–Whitney's *U* test. Of note, the numerical comparison of (C) and (D) is invalid because the conditions for protein trapping in the Formvar membrane differed between the two systems. (E) Negative‐staining EM images of PSDLs before contact with tubulin‐containing droplets. (F) Negative‐staining EM images of PSDLs after incubation with MTs subjected to the polymerization–depolymerization cycle. Clearly visible meshwork structures are indicated by arrows.

A schematic of the experimental design used to evaluate the interaction between PSDLs or PSDs and polymerizing MTs is shown in Figure 1A. PSDLs or the PSD protein was immobilized on the Formvar membrane‐covering EM grid by placing the grid on a PSDL‐ or PSD‐containing droplet (3–4 μL; PSDL mean concentration: 129 ng protein/μL; OG12‐PSD, approximately 160 ng protein/μL; TX‐PSD; 2.48 μg protein/μL) (Suzuki et al. 2021) formed on clean parafilm, and allowed to rest for 10–15 min at 0°C. Then, the Formvar membrane on the EM grid was incubated sequentially with TBS (20 mM, pH 7.5) containing 10% normal goat serum (NGS) for 10 min and TBS containing 5% BSA at 0°C for 3 min to minimize nonspecific binding of proteins at 0°C, and rinsed in GTB at 0°C. The Formvar membrane was further blocked with 10% NGS at 37°C for 15 min in case the grids were incubated thereafter at 37°C. This additional blockade is necessary to prevent nonspecific binding of polymerizing MTs to unblocked interior areas that may be exposed during tubulin polymerization at 37°C. The blocking effects are shown in Figure S1B. The NGS‐containing blocking solution contained a 1/100‐diluted protease inhibitor mix (Sigma‐Aldrich, St Louis, MO, USA), unless otherwise stated. Tubulin‐containing droplets were covered with an EM grid, with the PSDL/PSD‐laden side facing down on the droplet. Tubulin polymerization was initiated by transferring the dishes to a 37°C heat block. The dishes were surrounded by a humidified container to minimize evaporation during polymerization. At a specific time point, the grid was placed for at least 5 min on the PBS droplet containing 1% glutaraldehyde at room temperature. Polymerization was continued for the desired period or 30 min when the tubulin polymerization reaction reached equilibrium (within 20 min, according to the datasheet for tubulin, Cytoskeleton Inc. Denver, CO, USA). Then, the dishes were transferred onto ice after 30 min and allowed to rest for 25–40 min to induce MT depolymerization as excess MTs impede observation by EM; then, the samples were fixed with 1% glutaraldehyde at 0°C. Finally, the fixed samples were rinsed thrice with H_2_O and negatively stained with nano‐W (Molecular probes, Yaphank, NY, USA).

### 2.6 Experiments Using Latex Beads

To characterize the observed interaction between PSDL and polymerizing MTs, we investigated the binding of polystyrene latex beads (0.474 μm in diameter, NISSHIN EM Co. Ltd., Tokyo, Japan) to the polymerizing MTs. Two experimental approaches were employed with latex beads. First, PSDL was simply replaced by latex beads in the system used for the PSDL‐tubulin/MT interactions, where PSDLs were immobilized on EM grids. Latex bead‐laden grids were blocked with NGS and BSA at both 0°C and 37°C. ML116 tubulin (2 mg/mL) was polymerized at 37°C for 5–6 min.

In the second approach, tubulin polymerization was performed at 37°C in a small droplet (~10 μL) containing latex beads (final concentration of beads: 0.005% solids, in the same buffer used for the PSDL‐tubulin interaction). The beads were pretreated with 5% NGS at room temperature for at least 30 min to minimize nonspecific protein binding prior to incubation with tubulin (ML116, 2 mg/mL). The unblocked Formvar membrane‐covered EM grids were placed on the reaction droplet during tubulin polymerization for 5–6 min. As a control, the buffer containing beads was incubated under the same conditions, but in the absence of tubulin. Samples on the grids were fixed with 1% glutaraldehyde, washed with H_2_O, and stained with nano‐W.

### 2.7 Quantitative Analysis of PSDL‐MT Interaction and Bead‐MT Interaction

EM images were captured randomly at 6000–10000× magnification to quantify the contact of PSDLs with polymerizing MTs. Areas where MTs were densely distributed, making contact invisible, were excluded from quantification. PSDLs with longer diameters greater than 200 nm were investigated.

For identification and quantification of MTs associated with latex beads, EM images were captured randomly at 4000×–12,000× magnification.

To quantify the beads or PSDs (OG‐12) entrapped on the grids, EM images were captured randomly at a 600× or 8000× to 15 000× magnification, respectively, and the number of them was counted manually. A solution containing only latex beads or PSDs, without tubulin, was also incubated under the same conditions to serve as a negative control.

### 2.8 Evaluation of the Effects of MT‐Affective Reagents

PSDLs and PSDs were incubated at 0°C for 90 min in GTB containing various MT‐affective reagents and a protease inhibitor mix (1/50, Sigma‐Aldrich), transferred to a Formvar membrane on an EM grid, and then negatively stained with nano‐W. Samples without reagents were incubated in parallel as control specimens. GTP, GMP‐PNP, GDP, and ATP were used at a concentration of 10 mM; nocodazole was used at 10 μM, and paclitaxel at 1 μM. The 10 mM concentration for GTP, GMP‐PNP, GDP, and ATP was chosen to enable clear detection of morphological changes in the ultrastructure. The effects of treatment with 1 mM GTP were also confirmed (Figure S5). DMSO (0.1%) was also used as the control treatment for experiments involving nocodazole and paclitaxel, as stock solutions of both reagents (10 and 1 mM, respectively) were prepared using DMSO, with both treatments containing 0.1% DMSO. The other reagents used in this study were water‐soluble, and their stock solutions did not contain organic solvents. The presence of the protease inhibitor mix had only a negligible effect on the morphology of the structures tested after incubation at 0°C for 90 min.

Two types of PSD preparations were used depending on the aim of the experiment. To evaluate changes in non‐aggregated PSD structures, OG12 was used because its purification from stored SPM is relatively easier than that of conventional PSD. Thus, OG12 can be easily prepared and stored unfrozen at −30°C in the presence of 50% glycerol for a relatively short period. Based on EM observations, the structure of OG12 was not altered for at least 1 year. OG12 under 6 months of storage was used for the core experiments in this study. To observe changes in PSD aggregation, TX‐PSD stored at −80°C was used. The protein profile of the TX‐PSD was similar to that observed immediately after purification, suggesting that no significant proteolysis occurred during storage.

To quantify the extent of damage caused by the reagents, we assessed the degree of structural changes in the remaining PSDL or PSD posttreatment, despite the limitation that extremely damaged fragmented into small, widely dispersed pieces were excluded from being counted. The evaluation of reagent effectiveness against PSDL and non‐aggregated PSD involved scrutinizing the specimens at both low (< 10 000‐fold) and high (> 15 000‐fold) magnifications. The primary objective of examination at low magnification was to know the vastness of the damage, and to identify the pervasive structural deteriorations that led to the almost complete obliteration of the original configurations, causing the dispersion of smaller fragments. A high magnification examination was necessary to determine whether the remaining original structures were damaged, thereby strengthening the identification of the damage observed at low magnification. EM images of PSDL and OG12 structures posttreatment with diameters ranging from 150 to 1500 nm were taken at high magnifications (typically greater than 25 000×). Structures less than 100 or 150 nm in diameter may include fragments that were produced during purification and present before the treatments. Structures larger than 1000 nm, in particular PSDs, may be aggregated ones. Thus, structures with a diameter of 150–1500 nm were counted. The morphologies of the remaining PSDL structures posttreatment were categorized into three groups: normal to nearly normal (N), moderately damaged (M), and severely damaged (S). For the remaining PSDs posttreatment, the morphologies were classified into two groups: normal to moderately damaged (NM) and severely damaged (S), as distinguishing “M” from the others was more challenging in PSDs compared to PSDLs. Severely damaged structures included those with many dispersed fragments and an extremely broken overall shape, as well as those that maintained their overall shape but contained extensively modified internal components. Structures judged to be “M” maintained their overall shape. For quantification of the effects on the aggregated PSDs, EM images were captured randomly at a 200× magnification, and particle sizes and numbers in a single square field of a 400 mesh EM grid were determined through particle analysis using the ImageJ 1.53k software (for details, see experimental design).

### 2.9 Immuno‐Gold Negative Staining

Negative staining coupled with the immuno‐gold technique using 10‐nm gold particles was carried out as previously described (Suzuki et al. 2018; Suzuki et al. 2021). Primary antibody dilutions were determined based on data from a previous study (Suzuki et al. 2021). The antibody diluent contained a protease inhibitor mix (1/100, Sigma‐Aldrich). All samples were fixed with 1% glutaraldehyde in PBS for 5 min immediately after incubation with secondary antibodies. To evaluate the effects of GTP, we conducted a swift fixation using 0.25% glutaraldehyde immediately after treatment with GTP. This fixation step was essential before incubation with primary antibodies to avert the inadvertent loss of short fibers observed during immuno‐gold negative staining.

### 2.10 Electron Microscopy

Specimens were examined using a JEOL JEM‐1400Flash (JEOL Tokyo, Japan) at 80 kV at different magnifications (200–120 000×), and images were captured using a 4008 × 2672‐pixel element CCD camera (Gatan SC1000; Gatan Inc., Pleasanton, CA, USA). The contrast of the images was edited using Photoshop Elements 9 (Ver. 20.0) (Adobe Systems Inc., Singapore) to clearly visualize the gold particles, without modulating the γ‐Contrast. The smart correction function in Photoshop Elements 9 was used to improve morphological obscurity due to darkness encountered during the examination of the negatively stained specimens. A slight change in contrast can significantly affect the negative staining image, making proper contrast particularly crucial for the identification of PSDLs. The Formvar membrane attached to the EM grid was subjected to a wetting treatment by Hydrophilic treatment device HDT‐400 (JEOL Tokyo, Japan), and the wetted EM grid was used within 1 week.

### 2.11 Biochemical Assays

SDS‐PAGE was carried out using a 10% polyacrylamide gel. Proteins were stained with Coomassie brilliant blue (CBB)‐R250. Protein profiles were captured by a WSE‐5200 Printgraph 2M (ATTO Bioscience & Technology, Tokyo, Japan). The electrophoresis images have not been rearranged or spliced. The contrast was adjusted using Photoshop for clarity. Protein profiles were acquired under unsaturated conditions, and the areas of the tubulin bands were quantified using ImageJ, an open‐source platform for biological‐image analysis (Schneider et al. 2012).

For the binding assay, tubulin (T240 or M116, 5 μg) was mixed with OG12 (approx. 1 μg) in 20 μL of GTB and incubated at 37°C for 20 min. The samples were then centrifuged at 12 000*g* for 90 s at room temperature to sediment the PSDs. The resulting pellets were washed once with 20 μL of GTB. Subsequently, SDS‐PAGE followed by CBB‐250 staining was performed on the pellets to detect the amount of tubulin bound to the PSDs.

### 2.12 Experimental Design and Statistical Analysis

Male rats were used for synaptic subfraction purification to exclude sex differences. The specificity of the immune reaction in the immuno‐gold experiment was verified using control specimens processed without primary antibodies.

For quantitative analyses, EM images were captured randomly at the magnifications shown in the corresponding figure legends. Particle size (area, maximum, and minimum diameters) and numbers in a single square of a 400 mesh EM grid obtained from imaging at 200× or 600× magnification (a square with one side length approx. 44 μm, Nisshin EM Co. Ltd., Tokyo) were measured through particle analysis using the ImageJ 1.53k software (Schneider et al. 2012), unless otherwise stated. Particle identification was carried out based on criteria established using the optimal fitting algorithm for each specimen. Additional individual scrutiny was carried out on particles located along the periphery of the grid through a meticulous manual assessment. Small particles, such as those less than 100 nm in maximum diameter, unless otherwise stated, were not counted for PSDL or PSD quantitation as most of these particles had ambiguous identities or were possibly damaged. The number of latex beads was manually counted on the enlarged, brightness‐adjusted EM images displayed on a PC monitor. This adjustment was necessary to distinguish the small dark spots containing beads from those without. The presence and quantity of beads in the dark spots were easily and accurately identified in the EM images after a slight enhancement of the brightness.

Quantitative data are presented as the mean ± standard error (SE). Statistical analyses (Shapiro–Wilk normality test, Student's *t* test, and Mann–Whitney's *U* test) were performed using GraphPad Prism 6.0 (GraphPad Software, San Diego, CA, USA). A Student's *t* test or Mann–Whitney's *U* test (both unpaired two‐tailed) was applied based on the distribution's normality, unless otherwise indicated. Results were considered statistically significant at *p* < 0.05. *p*‐Values and sample numbers are shown in each figure. No test for outliers was conducted. Full statistical reports can be found in the Supporting Information.

## 3 Results

### 3.1 Survey on the Interaction of the PSDL Structure With MTs In Vitro

We hypothesized that the interaction of the PSDL with tubulin or MTs that are present outside the PSD occurs since tubulin is by far the most abundant PSDL component (Suzuki et al. 2021) and MTs transiently intrude into the spine where PSDs are located. To identify the possible interactions, we used an in vitro system in which tubulin polymerization was induced in the presence of PSDLs and searched for interaction sites through EM observation. Isolated PSDLs were immobilized on a Formvar membrane attached to an EM grid. The PSDL‐loaded membrane was then blocked with albumin and NGS to minimize nonspecific protein binding, followed by exposure to a tubulin‐containing droplet and incubation at 37°C to promote MT polymerization (see Methods and Figure S1A). To assess potential changes in the PSDL structure during and after incubation with polymerizing MTs, the MTs were depolymerized at 0°C for 30 min. This system is advantageous in the situation where the PSDL protein concentration cannot be increased to that of tubulin due to the low yield of PSDL protein.

An overview of the interaction between PSDLs and polymerizing MTs, along with a control experiment in the absence of PSDLs conducted under the same conditions but using an unblocked EM grid membrane instead of a blocked one, is shown in low‐magnification images (Figure 1A,B). Electron microscopy of negatively stained PSDL preparation, prior to mixing with tubulin, revealed a dispersion of electron‐dense particles of various sizes (Figure 1A‐a), which were presumed to represent different sizes of PSDLs. The tubulin preparation used for the interaction also displayed dispersed small particles (Figure 1B‐a), most of which were clearly structurally distinct from the PSDL, as confirmed by enlarged views. Low levels of MTs were observed to be trapped in the membrane 5 min after the initiation of polymerization, while a higher abundance of MTs was trapped at 30 min (Figure 1A‐b,c, respectively). These MTs were primarily associated with PSDLs immobilized on the membrane. In contrast, MTs observed on the control unblocked grid membrane (Figure 1B‐b,c) resulted from nonspecific interactions with the membrane. Following depolymerization for 30 min, the trapped MTs were lost from the Formvar membrane as expected (Figure 1A‐d).

The size and number of PSDLs remained unchanged after incubation with polymerizing and subsequently depolymerized tubulin (Figure 1C), as did the particles in the tubulin preparation (Figure 1D). Most of the amorphous fragments derived from depolymerized MTs (indicated by thin and thick arrows in Figure S1C) disappeared after 30 min of cooling on ice, leaving only a few residual amorphous structures (open arrows in Figure S1D), which differed from the PSDLs. PSDL structures maintained their characteristic meshwork‐like configuration after interaction with polymerizing MTs (Figure 1E,F). Thus, changes to PSDL structures were minimal. The subtle alterations observed may be attributed to protease‐induced damage during long‐term incubation at 37°C, despite the presence of a protease inhibitor cocktail in the blocking solution.

**FIGURE 2:**
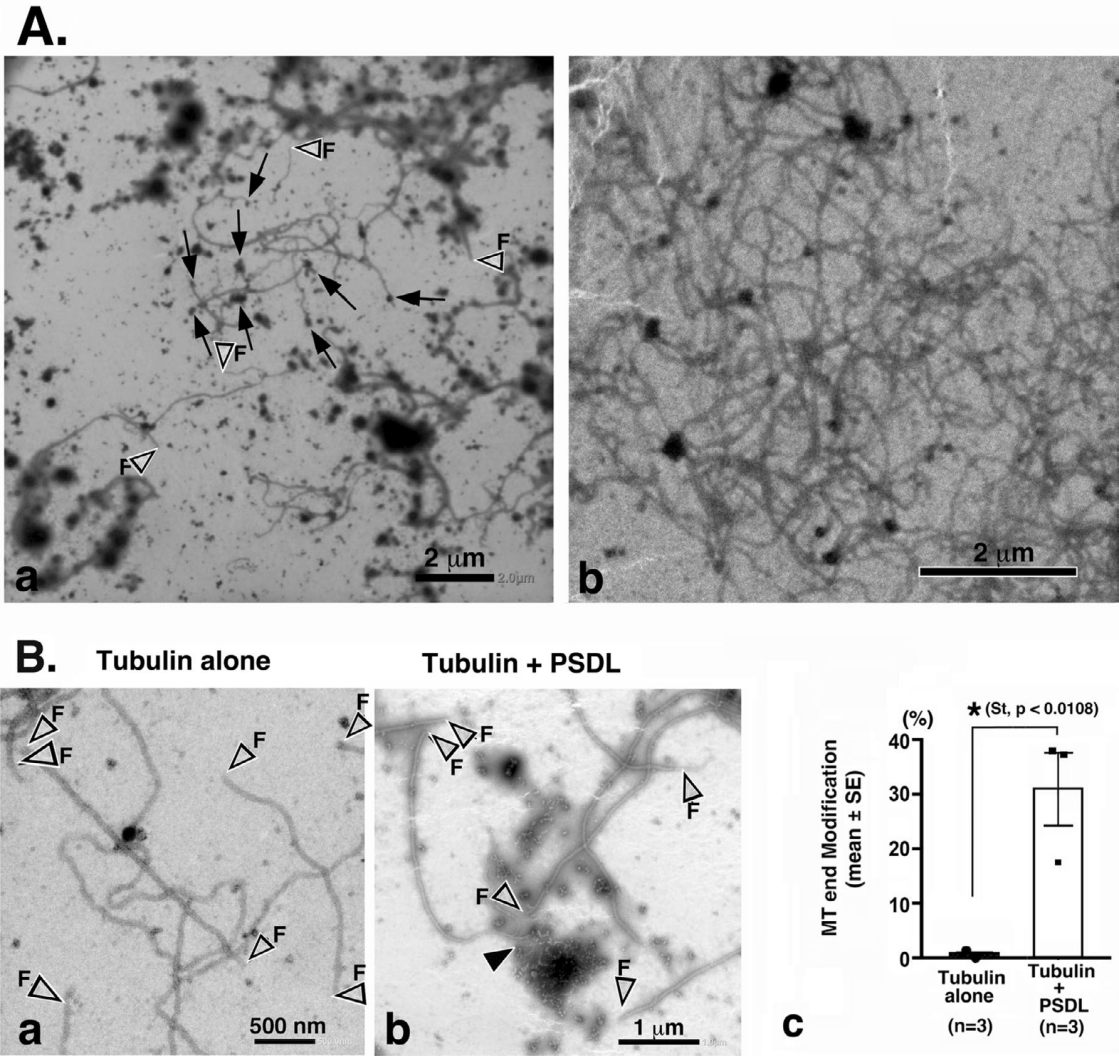
Interaction of PSDLs with MT ends. (A) Representative EM images clearly showing early stage interactions between polymerizing MTs and PSDLs. Both EM images were taken after MT polymerization at 37°C for 5 min. The polymerization stage was more advanced in (A‐b) than in (A‐a), as inferred from the length and the prevalence of polymerized MTs. Of note, relatively short MTs, possibly those just starting to elongate, were frequently associated with PSDLs in (A‐a). Some MT ends connected to PSDL particles and those unconnected are indicated by arrows and open arrowheads marked with F, respectively, in (A‐a). PSDL particles unconnected to MTs were widely distributed during the early stage of MT polymerization (A‐a). During the later stage (A‐b), the MT network, which connects PSDLs, significantly developed. (B) Identification of the interaction between MTs and PSDLs at MT ends. MT ends were observed 5 min after the induction of MT polymerization in the absence (B‐a) or presence (B‐b) of PSDLs. Free MT ends and PSDL‐associated MT end portions are indicated by open arrowheads marked with F and closed arrowheads, respectively. The contact portion indicated by the arrowhead in (B‐b) is magnified in Figure 5D‐a,b. (B‐c) Quantitation of MT ends associated with PSDLs or PSDL‐like structures. Polymerization was carried out for 5–10 min in a droplet that was covered with a PSDL‐laden EM grid or an unladen EM grid. The PSDL‐laden Formvar membrane was blocked at both 37°C and 0°C, but the PSDL‐unladen membrane was not blocked or blocked only at 0°C. Negative‐stained EM images were randomly taken at a 15 000× magnification, and both the free and PSDL‐associated MT ends were counted. The percentage of the PSDL‐associated MT ends was calculated from data obtained from three independent experiments, which included 179, 55, and 155 MT ends in the “tubulin alone” samples, and 162, 119, and 169 “tubulin+PSDL” samples. Significance was determined using Student's *t* test (two‐tailed).

The presence of MTs on the PSDL‐loaded grid membrane suggests an interaction between PSDLs and polymerizing MTs. We then investigated to determine the presence or absence of an association between PSDLs and polymerizing MTs through EM during the early phase of polymerization (a few to several minutes after initiating MT polymerization). The extent of MT polymerization varied, even for samples on the same Formvar membrane. Particularly, during the early stages, there were sparse areas with shorter MTs and relatively dense areas with longer MTs on the same Formvar membrane. The former may have occurred during the very early stages of MT polymerization. The observation of such early stage areas revealed a significant association between short nascent MTs and PSDLs (Figure 2A‐a). Thereafter, the number and length of MTs increased, with the maintenance of their association with PSDLs (Figure 2A‐b). These results suggest an interaction between PSDLs and polymerizing MTs.

Typical examples of MT ends formed in the absence or presence of PSDLs are shown in Figure 2B‐a,b, respectively. Most MT ends were free of any structures when polymerized in the absence of PSDLs (Figure 2B‐c). However, when MTs were polymerized in the presence of PSDL, approximately 30% of the MT ends were associated with structures including possible PSDL and PSDL‐like structures when observed 5 min after the initiation of polymerization (Figure 2B‐c). The proportion of MT ends associated with PSDL and/or PSDL‐like structures in the presence and absence of PSDLs in the total MT ends counted was 30.93 ± 6.68% and 0.77 ± 0.50% (mean ± SE), respectively (Figure 2B‐c).

### 3.2 Structures Similar to but Distinct From PSDLs Were Present in the Purified Tubulin Preparations

Based on the presence of MTs on the PSDL‐loaded grid membrane (Figure 1) and the increased modification of MT ends in the presence of PSDLs (Figure 2), we further investigated this modification in greater detail using electron microscopy at higher magnification.

**FIGURE 3:**
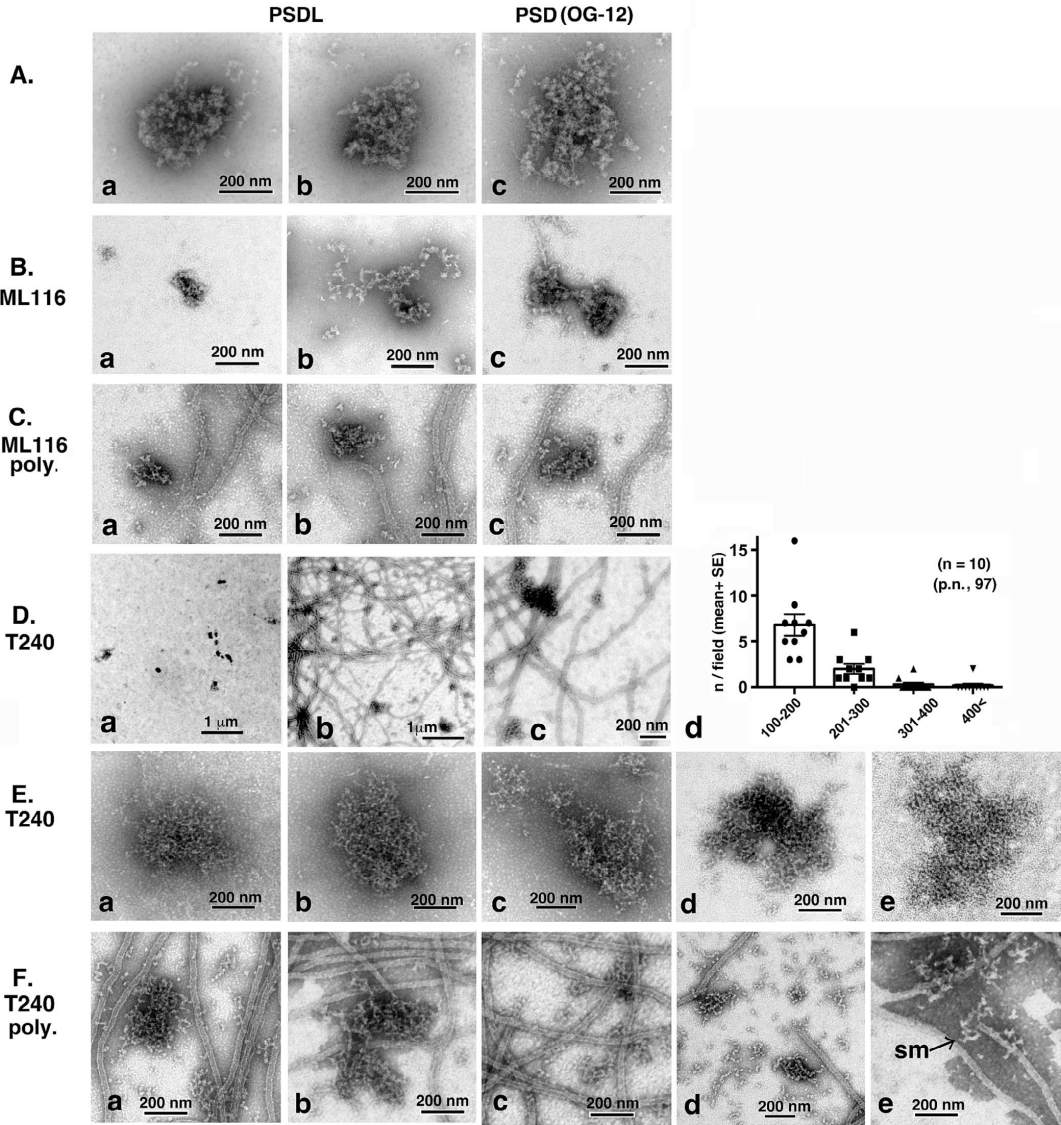
PSDL‐like structures contained in purified tubulin preparations. Examples of standard structures of PSDLs (A‐a and A‐b) and PSD (OG12) (A‐c) examined by negative‐staining EM. (B, C) Structures in MAP‐rich tubulin (ML116) before (B) and during polymerization at 37°C for 5 min (C), examined by negative‐staining EM. (D) Negatively stained pure tubulin (> 99% purity, T240) before (D‐a) and during polymerization at 37°C for 15 min (D‐b,c). The size distribution of the particles in T240 tubulin is shown in (D‐d). The vertical line represents the number of particles in a field imaged at 8000× magnification. The number of 8000× magnified images (n) and the total number of particles (p.n.) are indicated in the graph. Particles with a maximum diameter exceeding 100 nm were counted. (E, F) Negatively stained structures contained in the T240 tubulin before (E) and during polymerization at 37°C for 15 min (F). Both tubulin preparations labeled as “before polymerization” were suspended in the same buffer used for polymerization. All samples were fixed with 1% (v/v) glutaraldehyde prior to negative staining. An arrow labeled “sm” in (F‐e) indicates a small structure at the MT end (see also Figure S2 for enlarged images that better illustrate their morphological differences).

Identification of PSDLs in this study is based on their similarity to the electron micrographs of purified PSDLs published previously (Suzuki et al. 2018; Suzuki et al. 2021). The purified PSDLs observed by negative staining EM appear to be molecular assemblies with numerous tiny, hair‐like protrusions, which collectively form a mesh‐like structure inside. No specific structure is localized in any particular area. The diameter of the purified PSDL structures is approximately 300 nm. The negatively stained structure of purified PSDLs resembles those of isolated PSDs but is generally sparser than PSDs (Suzuki et al. 2018). Negative staining images of purified PSD have been reported by another group with structures similar to those presented in this study (Lai et al. 1999; Lo et al. 2007; Sui et al. 2000). The negative‐staining image of purified PSDs resembles the EM tomography images (Farley et al. 2015; Jung et al. 2023; Petersen et al. 2003; Swulius et al. 2010). Standard structures of PSDL and PSD observed in negative staining EM are typically displayed in Figure 3A, although the overall morphology of the purified PSDLs and PSDs shows variability.

Before a detailed investigation of PSDL‐related structures, we examined the structures contained in the M116 tubulin preparation by EM. We investigated the two types of tubulin preparations, MAP‐rich tubulin (ML116) and pure tubulin (T240), > 99% pure in SDS‐PAGE, according to the manufacturer's datasheet (see also Figure 10A [both from Cytoskeleton Inc., Denver, CO, USA]). This examination is necessary because the M116 tubulin preparation contained small particles (Figure 1B‐a,D). The particles in the ML116 tubulin were amorphous, with some forming a mesh‐like configuration, and mostly smaller than 200 nm (Figures 1D and 3B). Structures with fine meshworks were present before and after the induction of MT formation (Figure 3B,C). The T240 tubulin preparation also contained structures composed of fine molecules (Figure 3E,F). It contained a slightly higher proportion of 200 to 300 nm‐sized structures compared to the M116 tubulin preparation (Figure 3D‐d). These fine meshwork structures resemble PSDLs in overall appearance (Figure 3E). However, importantly, their internal structures differ (Figure 3; Figure S2). Apparent PSDL‐like structures were more frequently observed in pure tubulin preparations and were typically larger than those found in MAP‐rich tubulin. The structures present in T240 tubulin before polymerization were retained during MT formation, along with additional structures (Figure 3) associated with the MTs. The broad distribution of tubulin immunoreactivity was confirmed in the PSDL‐like structures present in the pure tubulin preparation (Figure S2D).

### 3.3 EM Observation of the PSDL Structures During Incubation With Polymerizing MTs

We investigated the potential interaction between PSDLs immobilized on the EM grid and polymerizing MTs in the system described in Figure 1. We encountered difficulty in distinguishing genuine PSDLs from PSDL‐like structures derived from the tubulin preparation after mixing, in the observation using negative EM staining. This was although the number of PSDL‐like structures with a size similar to that of genuine PSDLs is low in the ML116 tubulin preparation used for the interaction study (Figure 1D), and therefore, the likelihood of transferring these structures to the observed specimen is very low. Some PSDL structure may have changed during MT polymerization. In this article, structures that resemble PSDLs in both morphology and size including the original PSDLs, in the PSDL‐loaded grid incubated with polymerizing MTs are collectively referred to as “PSDL‐like structures.”

**FIGURE 4:**
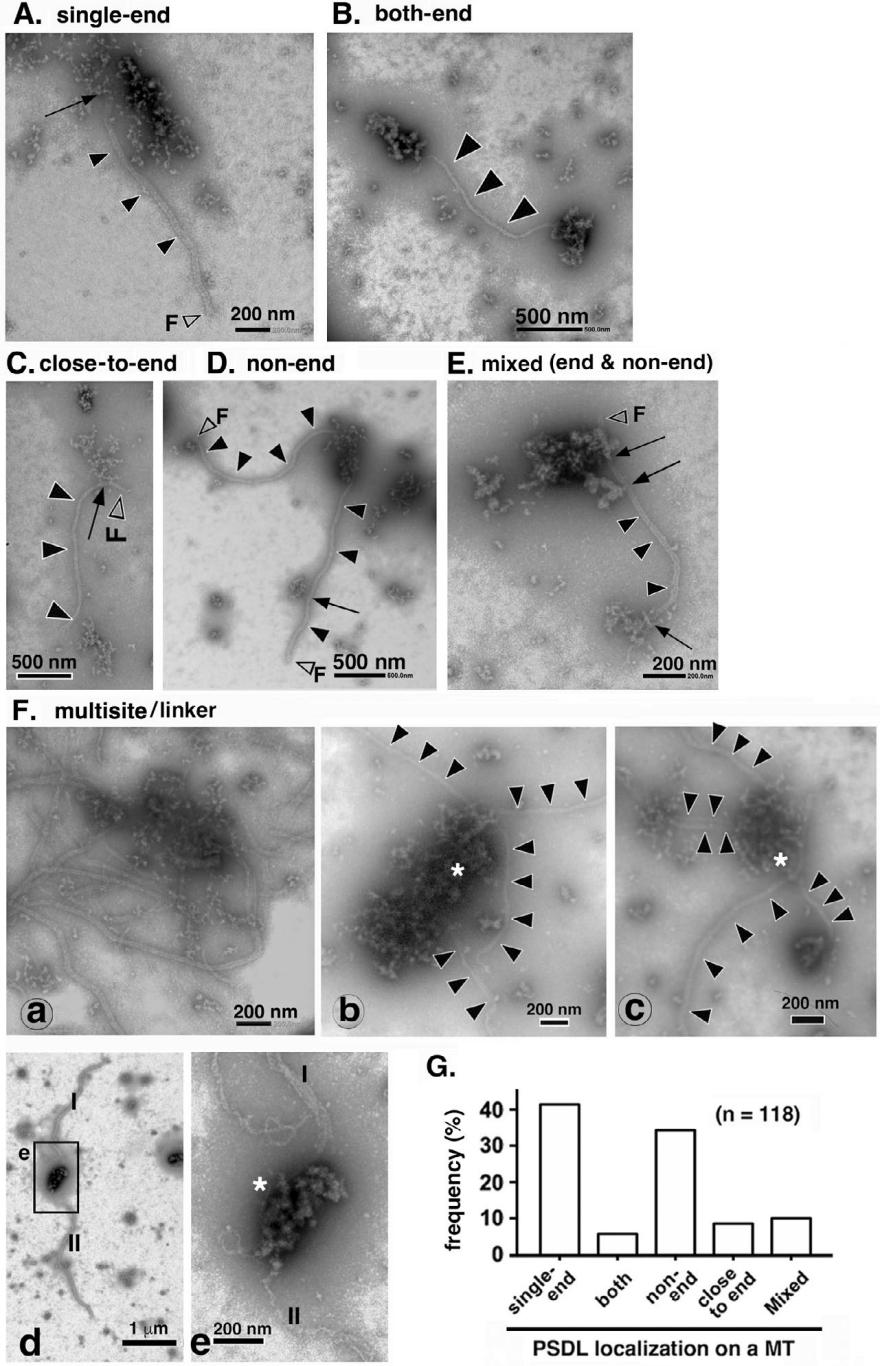
Distribution of PSDL‐like structures on MTs. Tubulin (ML116) was polymerized in the presence of PSDLs for 5–6 min as described in the legends of Figures 1 and 2. In this article, structures that resemble PSDLs in morphology and size, including the original PSDLs, are collectively referred to as “PSD‐like structures.” This is because distinguishing between them is extremely difficult. (A–F) Various types of associations between polymerizing MTs and PSDL‐like structures. To enhance clarity, MTs are indicated using closed arrowheads in low‐contrast photos. MTs are not labeled in (F‐a) because there are several MTs distributed in a complicated manner. Free MT ends are indicated with open arrowheads marked with F. Clearly identifiable contact sites between PSDL‐like structures and MTs are indicated with arrows. The image in (F‐e) is a magnified view of the rectangular region shown in (F‐d). PSDL‐like structures marked with asterisks connect MTs, particularly the one in (F‐e), which links MTs labeled I and II (F‐d and F‐e). The distribution frequency of PSDL‐like structures is shown in (G). The distribution of PSDL‐like structures on MTs was analyzed by capturing images of full‐length MTs at 6000–10 000× magnification and viewing the magnified images on a computer display. The frequency is presented as a percentage of the total PSDL‐like structures counted (118 PSDL‐like structures > 200 nm, as described in the Methods section). MTs exhibiting multisite‐type binding were excluded from the analysis due to the complex crowding of MTs. Structures distributed in the non‐end MT region, typically those smaller than 200 nm as indicated by the open arrow in (D), are also present in the tubulin preparation; therefore, they were excluded from this graph.

Associations between PSDL‐like structures and MTs were observed at various regions of the MTs. Representative images of these associations are shown in Figure 4. Some PSDL‐like structures were associated with only one end of the MT (single‐end type), both ends of the MT (both‐end type), or located at regions other than the ends (non‐end type), while others were found near the MT ends (close‐to‐end type). Additionally, multiple PSDL‐like structures and/or MTs were sometimes associated within a confined space (multiple‐type interaction). The frequency of PSDL‐like structure distribution on MTs is shown in Figure 4G. In this quantification, all PSDL‐like structures, regardless of their degree of resemblance to the standard PSDLs shown in Figure 3a, were counted.

**FIGURE 5:**
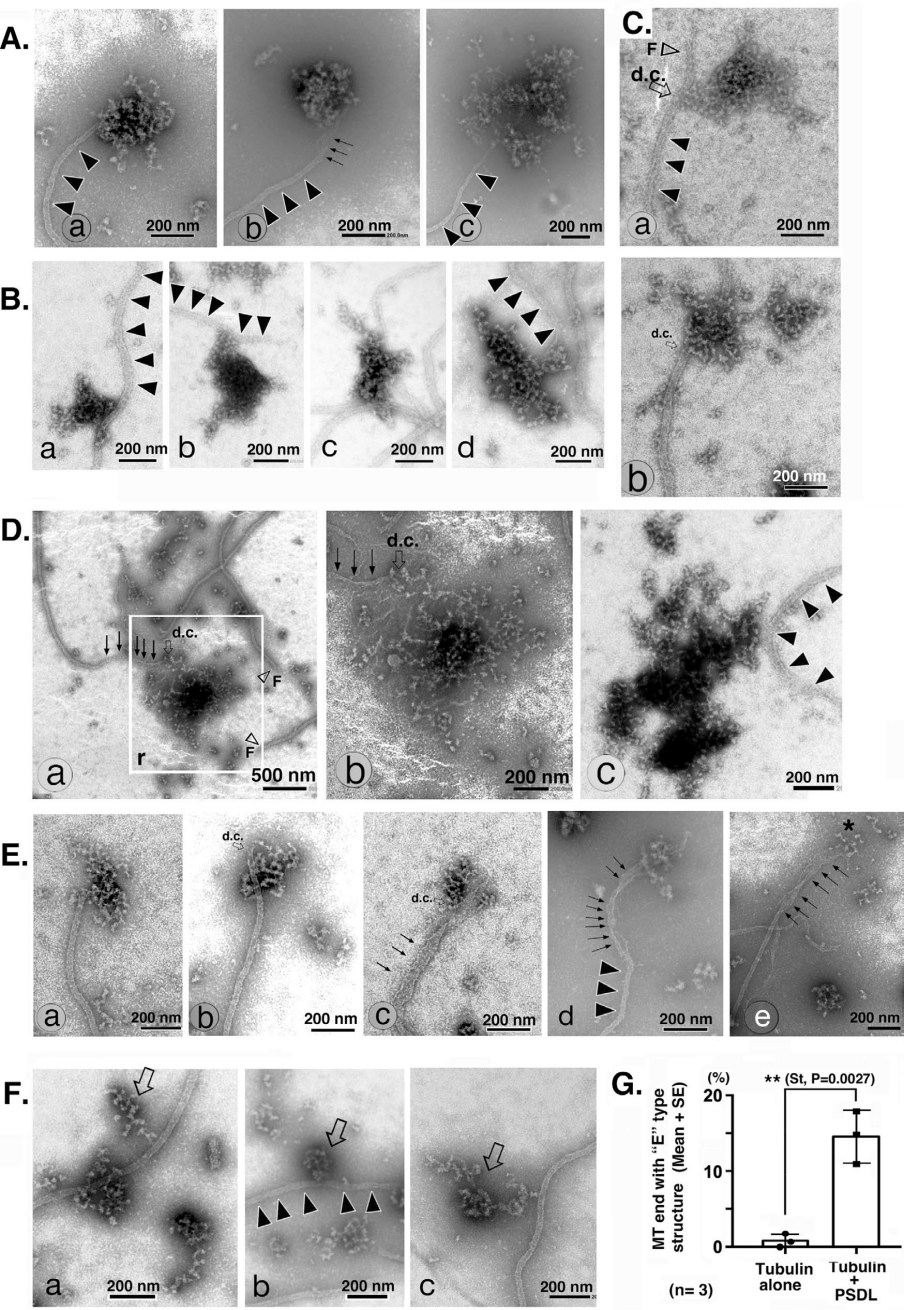
EM observation of contact points between polymerizing MTs and PSDL‐like structures. Tubulin (ML116) was polymerized in the presence of PSDL for 5 min, as described in the figure legends for Figures 1 and 2. Images focused on structures associated with MTs and their contact points between polymerizing MTs. The different types of associated structures and interactions are tentatively grouped and displayed in panels (A–F). Panel (G) shows the quantification of MT ends modified by associations with characteristic structures, as illustrated in panel (E). Samples were prepared, examined, and displayed as described in the legend for Figure 2B‐c. The percentage of PSDL‐associated MT ends represents the average of three independent experiments, which included 179, 55, and 155 MT ends in the “tubulin alone” samples, and 162, 119, and 169 MT ends in the “tubulin + PSDL” samples. Statistical significance was determined using a two‐tailed Student's *t* test. Direct connections (d.c.) between PSDL‐like structures and the MT end are indicated by small open arrows. Multiple arrows indicate tapered or enlarged MT end regions. Free MT ends are denoted by open arrowheads marked with “F”. MTs that are difficult to visualize due to low contrast are highlighted with multiple closed arrowheads. Characteristic mesh‐like structures associated with the MT non‐end regions are indicated by large open arrows (F). The images in panels (D‐a) and (D‐b) are magnified views of the lower half of Figure 2B‐b and the white square in panel (Figure 5D‐a), respectively.

The EM images shown in Figure 5 focus on the morphological variability of “PSDL‐like structures” associated with MTs, which are rarely observed during the polymerization of ML116 tubulin in the absence of PSDL (Figure 2C). Structural groups, each composed of structures with similar morphology, are presented in Figure 5. Clearly observable connecting portions are indicated by the small open arrows labeled with direct connection. The specimen examined contained various types of PSDL‐like structures with variable extent of resemblance to standard PSDLs. Structures with high similarity to standard PSDLs (Figure 5A) were connected to MTs at the end portion. Other types of structures similar to standard PSDL are associated at either MT end or non‐end portion (Figure 5B,C). Additionally, other meshwork‐like structures were associated with MTs at the MT end (Figure 5D‐a,b) or non‐end (Figure 5D‐c). The mesh structures in Figure 5C resemble those in Figure 5E, albeit the whole size is larger than the latter.

The structures in Figure 5E, particularly in Figure 5E‐d,e resemble a structure identified in the polymerizing MTs in pure tubulin preparation shown in Figure 3F‐e. They appeared to be generated from the MT ends as suggested by their appearance. This “E” group structures are very few in the MAP‐rich tubulin (ML116) in the absence of PSDLs even after starting their polymerization (Figure 5G), but the amount increased drastically by the presence of PSDLs during MT polymerization (Figure 5G). The result suggests the induction of the formation of these structures on the MT ends by the addition of PSDLs. The MT region close to the ends sometimes form an incompletely closed tubular structure but dilated or tapered in this group (small multiple arrows in Figure 5E‐c‐e as well as Figure 5A‐b). The small structures at the MT end (asterisk in Figure 5E‐e) may represent part of MT as similar structures were observed in pure polymerized tubulin (arrow marked with sm in Figure 3F‐e) and have been reported in the MT end regions during in vitro MT polymerization (Mandelkow et al. 1991).

There are unique meshwork structures associated with the non‐end portions of MTs (open arrows in Figure 5F). It is difficult to determine the identity of these PSDL‐like structures—whether they are genuine PSDLs or structures formed during tubulin preparation—because they are present on polymerizing MTs even in the absence of PSDL. These structures are typically small (less than 200 nm) in the absence of PSDLs; however, larger ones, such as those shown in Figure 5F‐a,c, are observed during MT polymerization in the presence of PSDLs, and their origin remains unclear. No further experiments were conducted to investigate this issue.

In summary, various types of PSDL‐like structures were found to associate with polymerizing MTs on EM grids, where immobilized PSDLs were incubated with polymerizing MTs. These associations were not observed prior to the mixing and incubation of the tubulin and PSDL preparations. Some of the MT‐associated structures correspond to PSDLs, while others appear to be structures formed on the polymerized MTs, like the structures shown in Figure 5E. The variability in the PSDL‐like structures may indicate modifications to the PSDL structure and tubulin polymerization, leading to the formation of the characteristic structures displayed in Figure 5. These results suggest an interaction between PSDL and polymerizing MTs.

### 3.4 Interaction Between OG‐Insoluble PSDs (OG12) and MTs In Vitro

The finding that the PSDL interacts with polymerizing MTs prompted us to examine the interactions of the PSD with polymerizing MTs as the PSDL is supposed to be the backbone structure of the PSD. Proof that PSD interacts with tubulin is more important than that between PSDL and tubulin in a physiological point of view because pure PSDL does not exist in vivo, but it exists as a PSD in a decorated form, except possibly during the early stages of PSD formation. The experiments here will provide key results for determining whether tubulin plays a role at the postsynaptic site.

The interaction of PSD with MTs was examined using the same system employed to determine the interaction of PSDLs with MTs, with PSDLs replaced with PSDs. We used two types of PSDs, the OG‐insoluble PSD (OG12) (Suzuki et al. 2021) (see also Materials & Methods), in which non‐aggregated PSD is abundant, and the Triton X‐100‐insoluble PSD (TX‐PSD), in which aggregated PSD is abundant.

**FIGURE 6:**
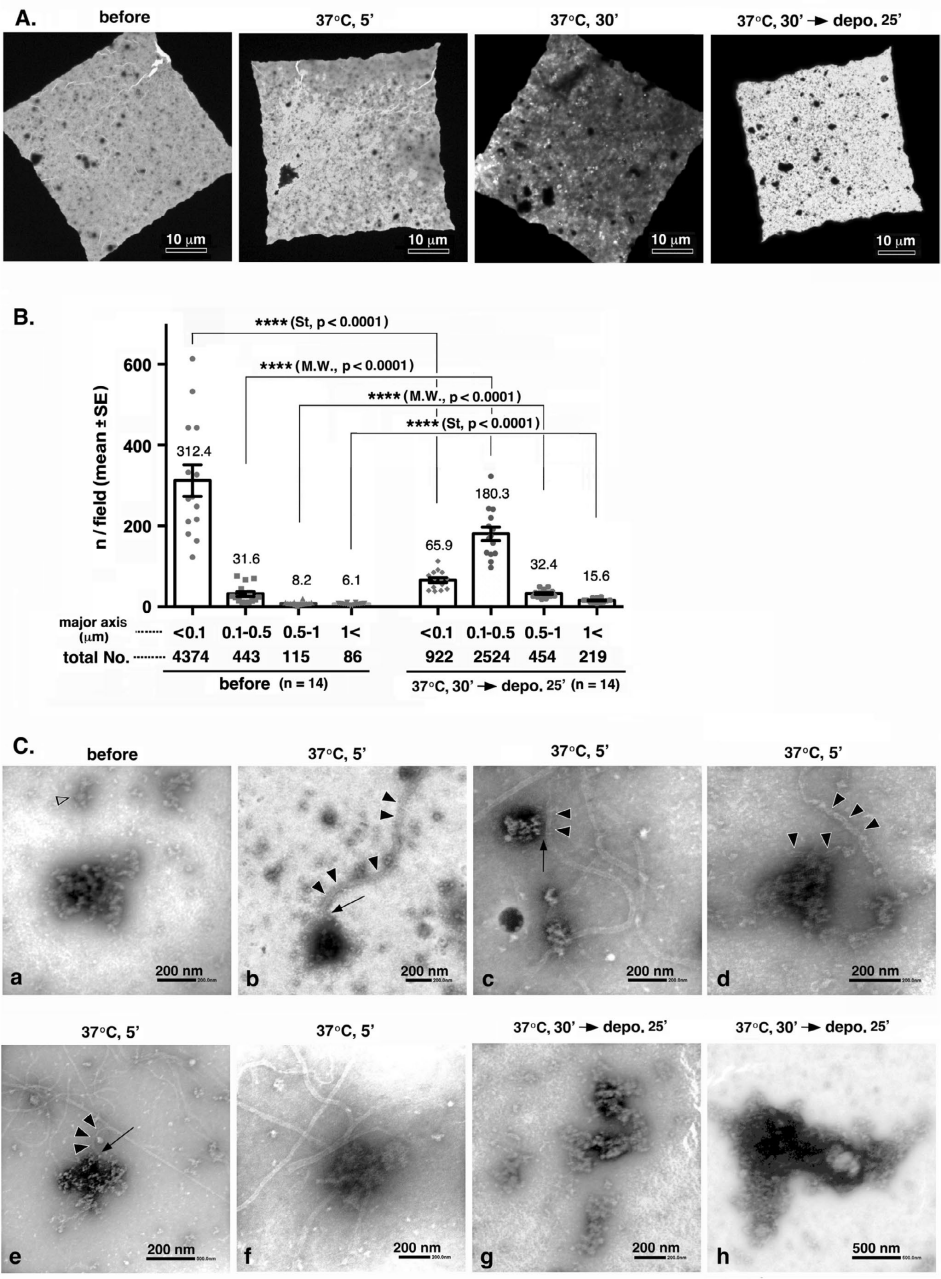
Interaction between polymerizing MTs and PSDs (OG12) in vitro. The interaction between PSDs and MTs was examined using the same system as that used for the evaluation of the interaction between PSDLs and MTs, with the replacement of PSDLs with OG12. (A) Time course of tubulin polymerization and subsequent MT depolymerization in the presence of OG12. EM images were captured at a 600× magnification. (B) Size distribution of particles as determined using negative staining EM of PSD‐laden membranes before and after incubation with tubulin subjected to the polymerization–depolymerization cycle. PSD‐laden membranes were blocked at 0°C and 37°C to prevent nonspecific binding, and then put in contact with the tubulin‐containing solution (ML 116, 2 mg/mL) during the polymerization–depolymerization cycle at 37°C and 0°C. Samples were negatively stained for EM. EM images were randomly captured at a 600× magnification, and particle sizes and numbers were determined through particle analysis using the ImageJ software. The number of areas counted (single square of a 400 mesh EM grid [Nisshin EM Co. Ltd., Tokyo]) was 14 for each sample, and the mean and total number of particles identified in each size group are indicated at the top and bottom of each bar, respectively. * Statistical significance. *P*‐values are presented in parentheses. St: Student's *t* test. (C) Morphology of negatively‐stained PSDs (OG12) before, during (37°C, 5′) and after (depo, 25′) interaction with polymerizing MTs. Interactions between polymerizing MTs and PSDs are observed in (C‐b‐f). Some MTs are traced with multiple closed arrowheads. Contact points between PSDs and MTs are indicated by arrows. The open arrowhead in (C‐a) indicates a typical small structure with an approximate diameter of 100 nm.

The time course of tubulin polymerization and subsequent MT depolymerization in the presence of OG12 is shown in Figure 6A. Particle sizes and numbers before and after incubation with tubulin undergoing the polymerization–depolymerization cycle were analyzed using ImageJ, and the results are shown in Figure 6B. The abundance of particles with maximum diameter below 100 nm significantly decreased, with a concurrent significant increase in the abundance of particles with diameter exceeding 100 nm, particularly those within the 0.1–0.5 nm range. A maximum diameter below 100 nm is smaller than that of intact mature PSDs; however, structural similarities, indicated by the open arrow in Figure 6C‐a, suggest that most, if not all, PSDs were fragmented or they were very early structures in their infancy. Small fragments and amorphous structures originating from MTs (Figure S1C) were not observed in the specimen. Consequently, the increased particle count was most likely not attributable to the incomplete removal of depolymerized MTs. The constancy in total particle count before and after exposure to polymerizing MTs (5018 and 4119, respectively) implied a subsequent increase in particle size after incubation with tubulin undergoing the polymerization–depolymerization cycle.

There were no discernible alterations in the PSD internal structure (Figure 6C‐a,g,h), although the increase in particle size (Figure 6B) suggested the growth of PSD structures. However, the overall morphology of the presumed PSDs showed variability, and typical disc‐shaped‐derived circular PSDs or oval‐shaped PSDs were not prevalent. Rather than exhibiting a circular or oval configuration, PSDs frequently exhibited a complex morphology both before and after interaction with polymerizing MTs as shown in Figure 6C‐g,h. This complexity in morphology appeared to be unaltered following interaction with polymerizing MTs. A direct interaction between non‐aggregated PSDs and polymerizing MTs was observed (Figure 6C‐b‐f), and this was similar to that observed between PSDLs and polymerizing MTs.

### 3.5 Interaction Between TX‐PSDs and MTs In Vitro

**FIGURE 7:**
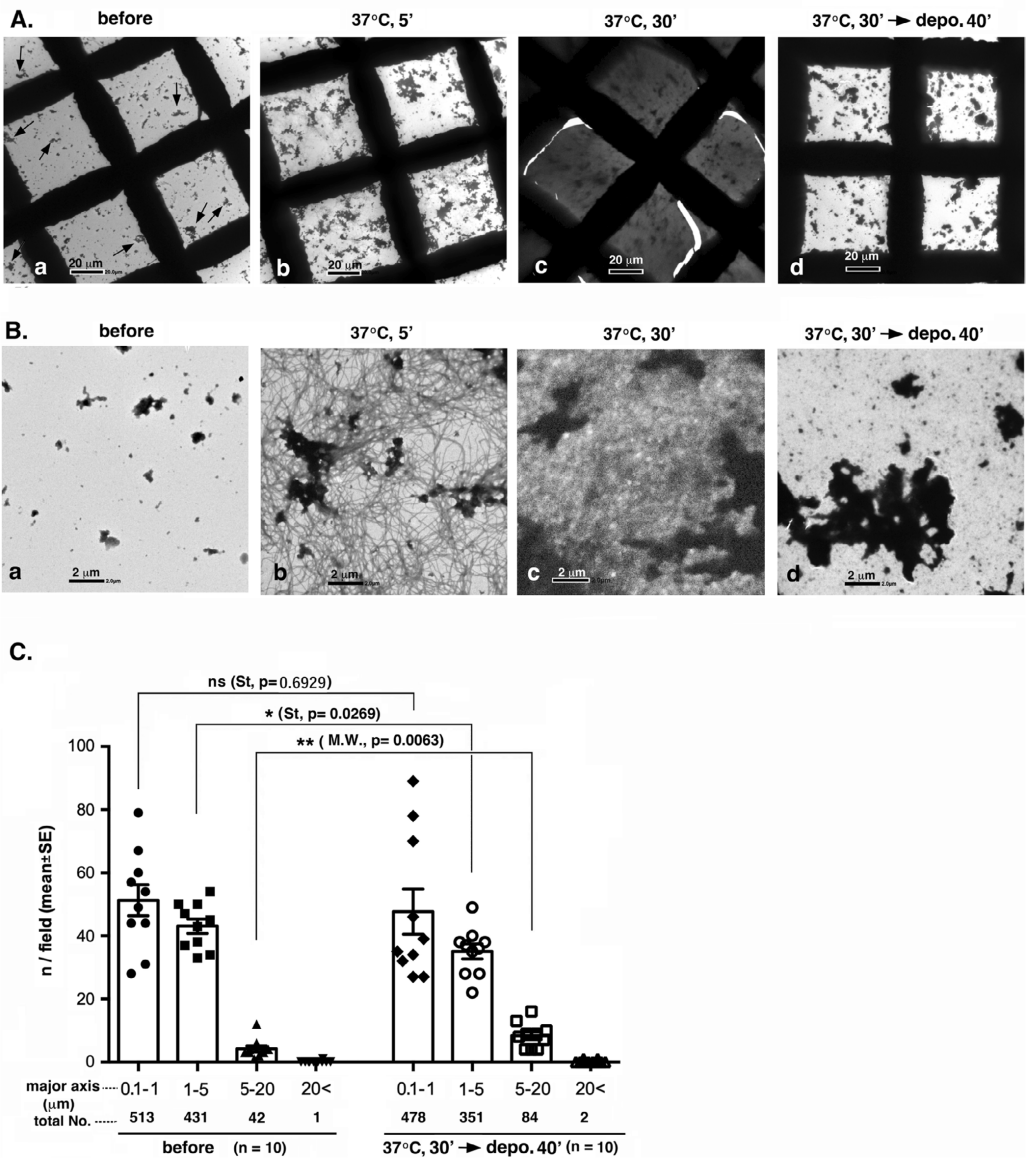
Interaction between polymerizing MTs and PSDs (TX‐PSD) in vitro. The interaction of PSDs with MTs was assessed using the same system as that used for the assessment of the interaction between PSDLs and MTs, with the replacement of PSDLs with TX‐PSD. Triton X‐100‐insoluble PSD (TX‐PSD) purified from rat forebrains through the conventional method (see Methods) was used. (A, B) Time course of tubulin polymerization and subsequent MT depolymerization in the presence of PSDs at different magnifications (200× and 2500× for A and B, respectively). Some PSD aggregates present in the preparation before interaction with polymerizing MTs are indicated using arrows (A‐a). (C) Samples were negatively stained for EM and particle analysis was performed as indicated in the legend of Figure 6. Ten areas were counted for each sample and the number of particles identified in each size group is shown at the bottom. * Statistical significance. *p*‐values are presented in parentheses. ns: nonsignificant; St: Student's *t* test. M.W.: Mann–Whitney's *U* test.

Next, the interaction between TX‐PSDs and polymerizing MTs was investigated. The time course of the interaction is shown in Figure 7A,B (low and high magnifications, respectively). The TX‐PSD preparation contained aggregated PSDs, typically with maximum diameter greater than 1 μm, even before contact with the tubulin‐containing solution (arrows in Figure 7A‐a). At the early stages of tubulin polymerization, for example, 5 min after the initiation of polymerization, the number and size of PSD aggregates increased (Figure 7A‐b). Large PSD aggregates were connected to a large number of MTs (Figure 7B‐b). Thirty minutes after incubation, all visual areas darkened, with an increase in the number of MTs, as shown in Figure 7A‐c and B‐c. After depolymerization at 0°C for 40 min, enlarged PSD aggregates persisted, but the surrounding MTs disappeared (Figure 7A‐d,B‐d). The quantification of structures on the EM grid revealed a significant decrease in structures with maximum diameters within the 1–5 μm range and a concurrent increase in structures with maximum diameters within the 5–20 μm range (Figure 7C). This observation implied a potential amalgamation of PSDs, with indications pointing toward the fusion of aggregated PSDs, and/or aggregated and non‐aggregated PSDs into more extensive PSD aggregates. The number of structures with maximum diameter within the 0.1–1 μm range, most of which should have contained non‐aggregated PSDs, did not change. However, their abundance might have increased, considering that the grid space unoccupied by the enlarged PSD aggregates was reduced.

**FIGURE 8:**
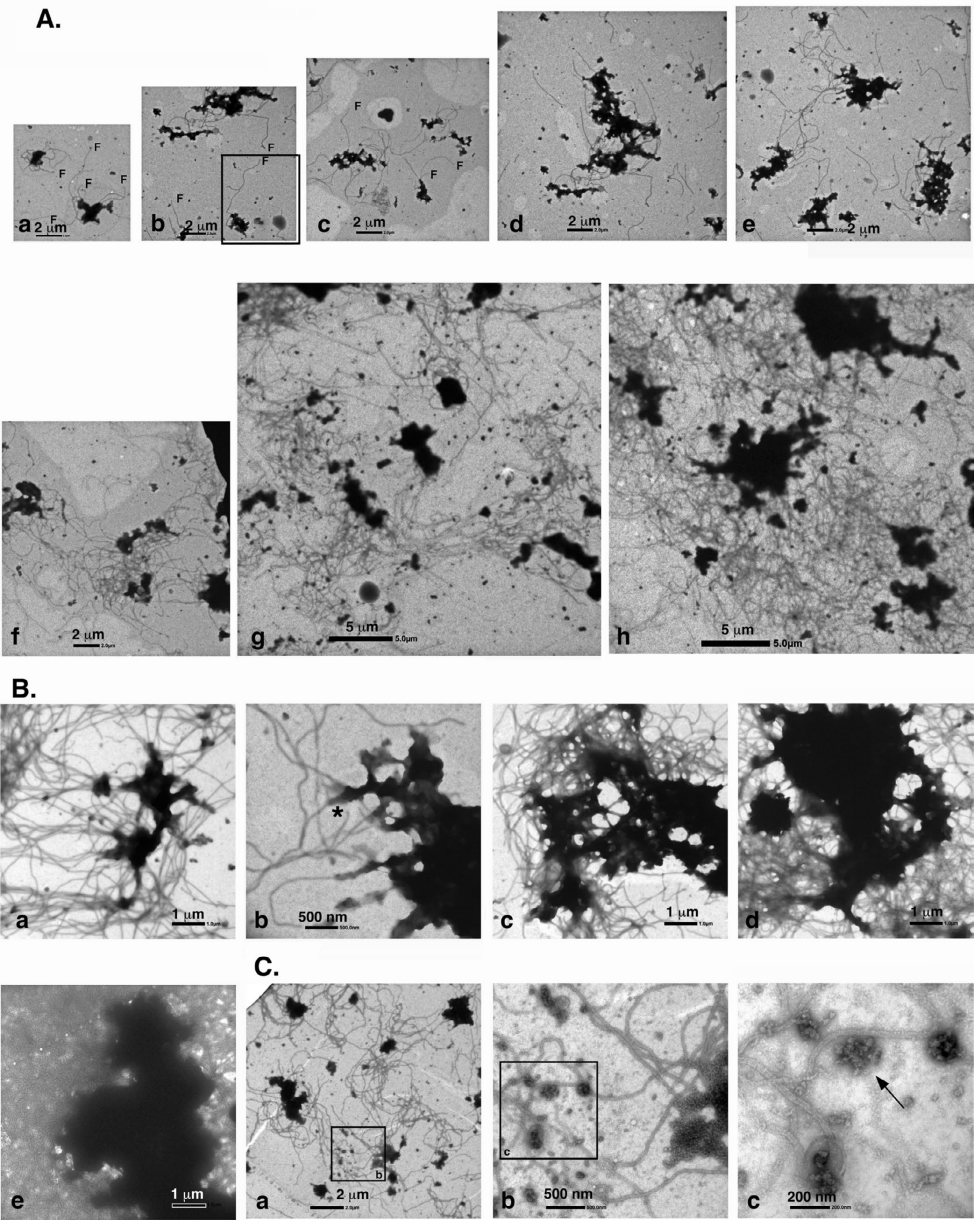
Representative EM images showing early stage interactions between polymerizing MTs and PSDs (TX‐PSD). The experiment was performed as indicated in the legend of Figure 7. Early stage interactions (5–9 min after the initiation of polymerization) between TX‐PSD and polymerizing MTs were observed through negative‐staining EM. (A) Images showing different extents of interaction between PSDs/PSD aggregates and polymerizing MTs. The extent of MT formation was variable, even within the same sample. The images are arranged in order of quantity from the least to the highest. All images are of the same scale. Typical free MT ends are indicated by F in (A‐a‐c). PSD aggregates in (A‐a) and those in the lower position (an area surrounded by a square) in A‐b are not connected, whereas those in the upper position in (A‐b) (also shown in A‐d) are connected by MTs. (B) Contact sites of MTs in PSD aggregates. Images of the contact sites were arranged in order of quantity from the least to the highest. The asterisk in (B‐b) indicates a typical contact site where MTs originate from different directions and form a bundle at the rim of the PSD aggregate. (C) Relationship between non‐aggregated PSDs and developing MTs. Images captured at low magnification. The boxed portions in (C‐a) and (C‐b) are enlarged in (C‐b) and (C‐c), respectively. The images in (B‐e) and (C) were captured 9 min after the initiation of polymerization, and the others were captured 5 min later. The arrow in (C‐c) indicates unaggregated PSDs.

The process of association between PSDs and PSD aggregates via MTs could be inferred in specimens at the early stage of MT polymerization, where MTs were sparsely distributed and variable in the extent of polymerization, even within the same sample. As shown in Figure 8A, the development of interactions with polymerizing MTs was more easily recognizable when images were arranged in order from that with the least to highest MT abundance. MTs connected to a single PSD aggregate exhibited free ends on the other sides, at least at the beginning (indicated by F in Figure 8A‐a,b,c). The images in Figure 8A‐a,b may depict the very beginning of the interaction. MTs connected to two or several PSD aggregates are shown in Figure 8A‐b‐e. The MT networks connecting the PSD aggregates gradually developed (Figure 8A‐f‐h). Figure 8B shows the commonly observed connecting points between the PSD aggregates and MTs, with the MTs forming bundles at the rim of the PSD aggregates. Typical contact points are indicated by asterisks in Figure 8B‐b. As shown in Figure 8B‐c‐e, during the late stages of MT polymerization, the PSD aggregates were heavily decorated with MT fibers, and the space surrounding them was filled with polymerized MTs. Non‐aggregated PSDs (e.g., the structure indicated by the arrow in Figure 8C‐c) were also connected to the MT fibers.

The association between PSDs and polymerizing MTs was observed using high magnification EM during the early stages of tubulin polymerization (5–9 min after the initiation of polymerization). The association observed between PSDs and MTs was highly similar to that between PSDLs and polymerizing MTs. Representative images have been incorporated into the supplementary data file (Figure S3). This association was detected both at the MT ends and non‐ends. In addition to the association between a single PSD and a single MT, an association was observed between a single PSD and multiple MTs. In some cases, MTs near the interacting PSDs were not completely tubular in structure (regions indicated by multiple arrows in Figure S3A‐c,d,e, D‐b,e). Interestingly, short MTs appeared like internal MTs in the PSDs (MT marked with arrowheads plus i in Figure S3D‐a). They appeared as bridges connecting two PSD‐like structures, as observed in fibers such as MTs; this is indicated with asterisks in Figure S3E.

**FIGURE 9:**
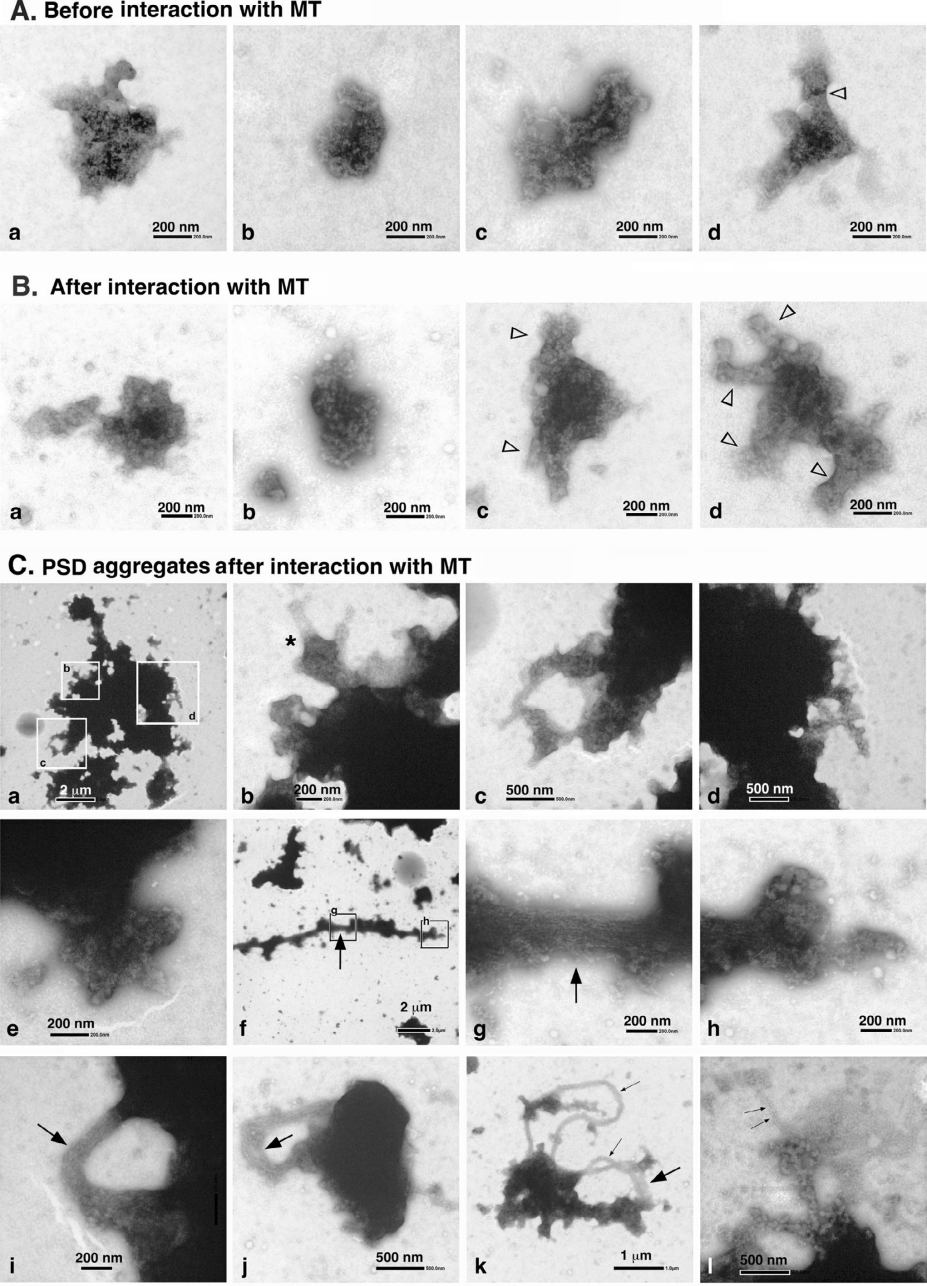
Changes in the PSD structure (TX‐PSD) following incubation with polymerizing MTs. Structures in TX‐PSD were observed through negative‐staining EM following incubation with polymerizing MTs and subsequent MT depolymerization. (A) Typical PSDs before contact with tubulin‐containing droplets. (B) Typical PSDs after the tubulin polymerization–depolymerization cycle. Protrusion‐like morphologies are indicated by the open arrowheads in (A‐d), (B‐c), and (B‐d). There appeared to exist an association between the two structures as shown in (B‐a). (C) PSD aggregates remaining after interaction with polymerizing MTs and subsequent MT depolymerization. The enlarged views of the white‐boxed regions in (C‐a) are enlarged in (C‐b), (C‐c), and (C‐d); the black‐boxed regions in (C‐f) are enlarged in (C‐g) and (C‐h). The asterisk in (C‐b) indicates a typical remnant of the contact site between MTs and PSD aggregates. The fiber bundles and thin fibers are indicated by the large and small arrows, respectively.

Our investigation focused on the alterations in PSD structures following their interaction with polymerizing MTs, as observed in negatively stained PSD specimens. Illustrative images of non‐aggregated PSDs within TX‐PSD samples are shown in Figure 9A,B. Similar to non‐aggregated PSDs within OG12 samples, a diversity of PSD morphologies was observed both before and after interaction with polymerizing MTs. PSDs exhibiting highly intricate morphologies were sporadically identified. Of note, some PSDs displayed protrusions (open arrowheads in Figure 9A‐d,B‐c,d), while others were interconnected (see Figure 9B‐a). This intricacy in morphology did not appear to change significantly following interaction with polymerizing MTs.

The morphology of the PSD aggregates persisted after the polymerization–depolymerization cycle as shown in Figure 9C. While most of the MTs surrounding the PSD aggregates disappeared, protrusions (asterisks in Figure 9C‐b) were observed at the rim region of the PSD aggregates. Fiber bundles and fibers associated with PSD aggregates (large and thin arrows, respectively, in Figure 9C), which may have been remnants of MT bundles associated with PSD aggregates during the MT polymerization period, were observed (compare Figures 8B‐b and 9C‐b). After the interaction, the PSD aggregates exhibited a significant number of such protrusions (Figure 9C‐a). These protrusions resemble those observed in the non‐aggregated PSDs (Figure 9B‐d). In addition, fiber bundles (Figure 9C‐i,j,k) and thin fibers (Figure 9C‐k,l) were associated with PSD aggregates. This finding indicated that certain MTs associated with PSD aggregates persisted even after MT depolymerization at 0°C possibly by slightly changing their biochemical properties. Consequently, this phenomenon may have contributed to an increase in the size of the PSD aggregates. Tubulin immunoreactivity within these fibers and fiber bundles was verified through immuno‐gold negative staining EM (Figure S4). Consequently, it was unequivocally established that the diverse array of fibers and fiber bundles linked to PSD aggregates were derived from MTs.

In summary, the outcomes of the experiments conducted using OG12 (Figure 6) and TX‐PSD (Figures 7, 8, 9) revealed that both non‐aggregated and aggregated PSDs exhibited an increase in their dimensions upon interaction with polymerizing MTs.

### 3.6 Investigation of the Interaction Between Tubulin/MTs and PSDL/PSD Using Non‐EM‐Based Methods

The interaction between PSDL and polymerizing MTs was presumed to be reasonably specific, as it occurred under conditions where nonspecific protein binding to the proteins on the grid membrane and to the membrane itself was effectively inhibited. This inhibition was achieved by blocking the PSDL‐laden membrane with NGS and BSA at both 0°C and 37^°C^ (see Figure S1B). To further verify both the specificity and robustness of the interaction between PSDL and polymerizing MTs, we employed non‐EM‐based approaches including co‐sedimentation analysis and experiments using latex beads.

**FIGURE 10:**
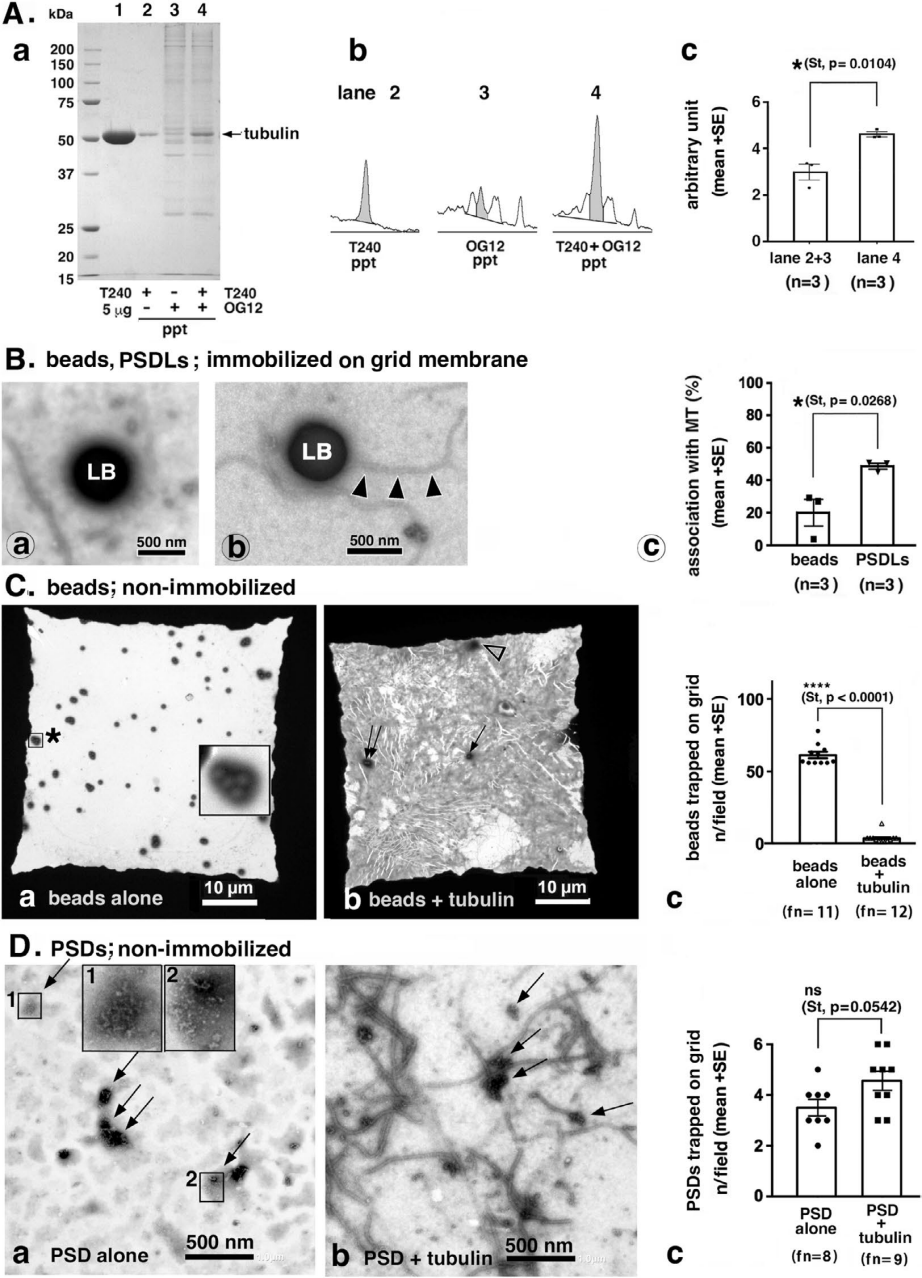
Investigation of the interaction between tubulin/MTs and PSDL/PSD using non‐EM‐based methods. (A) Co‐sedimentation analysis. Binding of tubulin to the PSD (OG12). Pure tubulin (T240, 5 μg) was mixed with OG12 (approx. 1 μg) and incubated for 20 min at 37°C. The samples were centrifuged, washed once, and the final pellets were analyzed by SDS‐PAGE (A‐a). Tubulin alone and OG12 alone were processed in the same way for comparison. The areas of the tubulin bands in lanes 2, 3, and 4 (gray) shown in (A‐b) were quantified, and the results are presented in the graph (A‐c). (B–D) Experiments using latex beads. (B) Entrapment of polymerizing MTs into immobilized latex beads. Latex bead‐immobilized EM grid was contacted to the solution in which MT (ML116) was polymerized. The grids were fixed, negative‐stained after 5 min of MT polymerization, and observed by EM. (B‐a, B‐b) Typical examples of the latex beads (LB) on the EM grids without or associated with MTs. Arrowheads indicate MT. (B‐c) The same experiment was carried out using a PSDL‐immobilized EM grid. Graph showing the amount of grid‐immobilized beads or PSDLs associated with MTs. The bars show mean values (mean ± SE) of three quantifications, which included 85, 108, and 75 beads, and 310, 355, and 305 PSDLs. (C, D) Tubulin (ML116) and latex beads (C) or PSD (D) were mixed in a solution, and MT polymerization was induced at 37°C for 5 min. They were captured on an unblocked EM grid, negatively stained, and observed using electron microscopy (EM) at 600× magnification. Beads alone were incubated and treated in the same manner as a control. The number of beads trapped on the grid was counted manually, and the quantification results are presented as the number of beads in a single square of a 400‐mesh EM grid (C‐c). Most dark spots in (C‐a) contained a single bead, while some contained clustered beads, as shown in the magnified area marked with an asterisk (C‐a). Only three beads are trapped in the field shown in (C‐b), as indicated by the arrows. The spot marked with an open arrowhead does not contain a bead. The dark background in (C‐b) is due to polymerized MT networks trapped on the grid. Tubulin (ML116) and PSD (OG12) were mixed in a solution, followed by the procedure described in (C), except for the EM imaging, which was performed at 8000× magnification. The field area in the graph corresponds to images obtained at this magnification. The number of entrapped beads was significantly reduced upon incubation with polymerizing tubulin, as shown in (C‐c), while the number of entrapped PSDs remained unaffected (D‐c). Arrows in (D) indicate PSDs, as confirmed by the enlarged views shown in (D‐a, 1, and 2). Statistical significance was assessed using a two‐tailed Student's *t* test, with the *p*‐value indicated in parentheses. fn, field number counted.

We confirmed tubulin (T240) binding to the PSD (OG12) by observing an increased amount of tubulin co‐sedimented with the PSD (Figure 10A). A similar result was obtained in our previous experiments using purified synaptic junctions, which showed that binding was both temperature‐ and concentration‐dependent and could be inhibited by MAP2 (Suzuki et al. 1987). In this study, we likewise observed a suppression of tubulin binding to the PSD when MAP‐rich tubulin (ML116) was used (data not shown); however, the underlying reason for this suppression remains unclear. We were unable to demonstrate that polymerizing MTs bind to the PSD, because under the tested centrifugation conditions (12 000*g* for 90 s and 100 000*g* for 30 min in 40% sucrose), MTs pelleted under the same conditions as the PSD. Therefore, sedimentation cannot distinguish whether MTs are bound to the PSD.

Next, we compared it to the interaction between polymerizing MTs and polystyrene latex beads. Two experimental approaches were employed for this comparison.

In the first approach, PSDL was replaced with latex beads in the system originally used for PSDL‐tubulin/MT interactions. In this experiment, we observed that MT association with the grid was suppressed due to blocking with NGS and BSA, although some MTs were still captured on the grid. The amount of MTs captured by the immobilized beads was significantly lower than that captured by immobilized PSDLs (Figure 10B‐c). This result confirms that the affinity of PSDLs for MTs is significantly higher than that of the latex beads.

In the second approach, tubulin polymerization was performed at 37°C in a small droplet containing either latex beads or PSDLs, maintained for 5–6 min. The content of the resulting sample was captured on an unblocked EM grid, and the number of captured beads or PSDLs was counted using negative staining EM. The amounts were then compared with those captured in the absence of polymerizing MTs. Due to the low protein concentration and limited availability of the PSDL stock, PSDs (OG12, see Methods for OG12) were used as an alternative in this experiment. In this experiment, the number of latex beads, but not of PSDs, captured on the EM grid was greatly and significantly reduced by the presence of polymerizing MTs (Figure 10C,D), although the beads were abundant in areas with heavy deposits of MTs on the grid, likely due to entrapment within the three‐dimensional network formed by the abundant MTs, given their low binding potency.

In both experiments using latex beads, we did not observe any structural modifications in the MTs. This suggests that the morphologies observed between PSDLs and polymerizing MTs (Figures 4 and 5) may result from a specific interaction between them. Collectively, these experiments support the conclusion that polymerizing MTs interact specifically with PSDLs.

### 3.7 Effects of MT‐Affective Reagents on PSDL Structures

On examining the interaction between PSDLs and polymerizing MTs, we did not carry out a negative control experiment without GTP as it is an indispensable reagent for in vitro tubulin polymerization. However, to the best of our knowledge, no studies have reported the effects of GTP on PSD structures. Therefore, we evaluated the effects of GTP on PSDL structures in a system that did not include tubulin polymerization.

Based on the results of pilot experiments (Figure S5), we treated samples with 10 mM GTP at 0°C for 90 min in the full‐scale experiment. We examined GTP analogs in the PSD and PSDL structures to determine the specificity of the effect of GTP. In addition, we examined the effects of nocodazole and paclitaxel, which are MT‐affective reagents. The experiments were carried out with or without the addition of a protease inhibitor mix to the incubation buffer. Changes in the PSDL structures were negligible after incubating samples at 0°C for 90 min, even in the absence of protease inhibitors.

**FIGURE 11:**
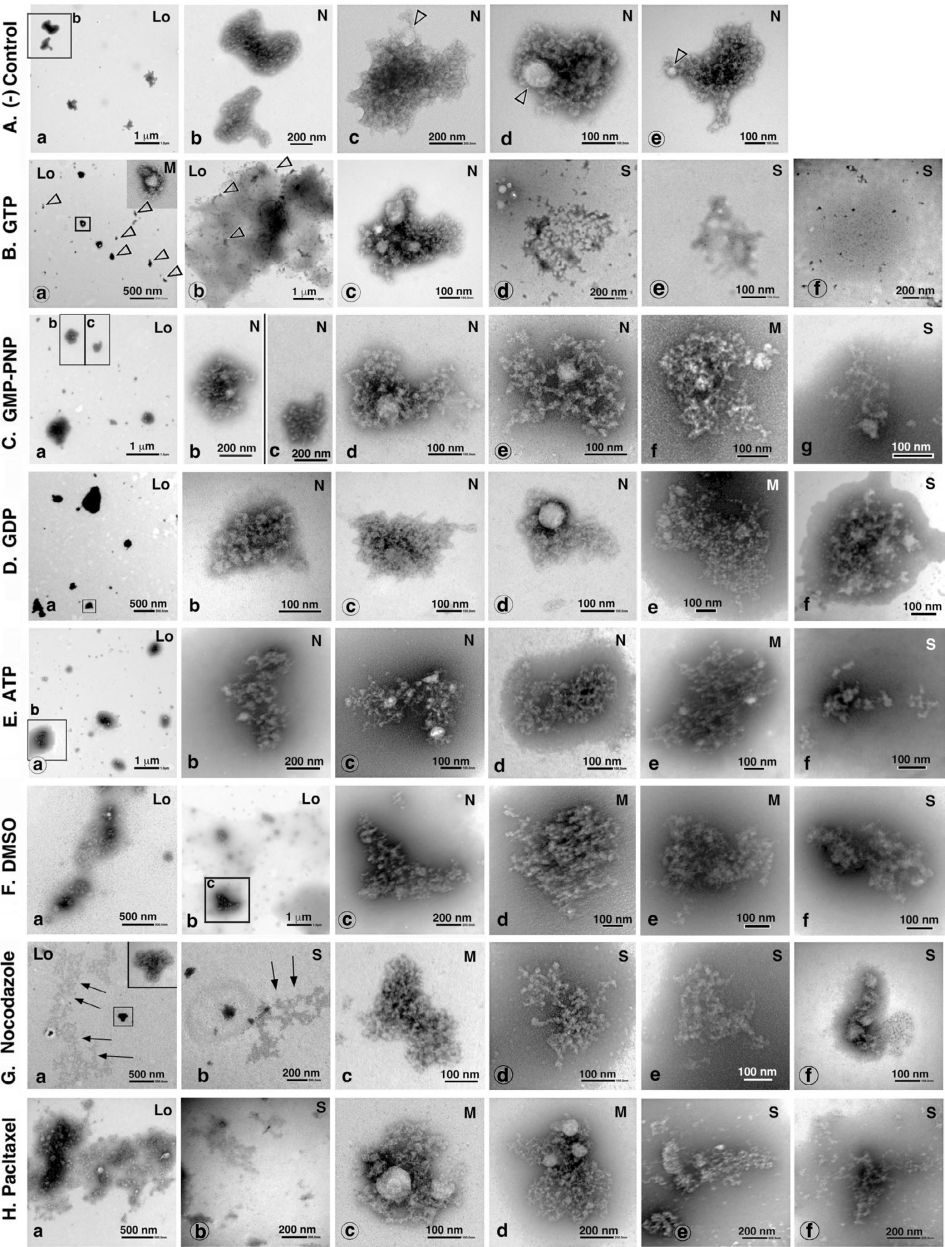
Effects of MT‐affective reagents on the PSDL structure. PSDLs were incubated at 0°C for 90 min in GTB containing various MT‐affective reagents, transferred to a Formvar membrane on an EM grid, and then negatively stained. The control sample was incubated without reagents at 0°C for 90 min. The nucleotide concentration was 10 mM, and nocodazole and paclitaxel were 10 and 1 μM, respectively. For treatment with nocodazole and paclitaxel, a buffer containing 0.1% DMSO was used as the solvent for these reagents. The effects of the reagents were analyzed for both low‐ and high‐magnification EM images. The low‐magnification images are marked with Lo. Morphology of the PSDLs after the reagent treatment was categorized into severely damaged (S), normal to nearly normal (N), and less extensively modified (M). Representative images are shown. The boxed portions of the structures observed at low magnification are enlarged in the same images or the adjacent right‐hand images. The enlarged images of (A‐a), (C‐a), (E‐a), and (F‐b) are normal, but that of (B‐a) suggests damage to the PSDLs in the small scattering spots produced after treatment with 10 mM GTP. Some of these small spots are indicated by open arrowheads in (B‐a) and (B‐b). Globular structures are indicated by open arrowheads in (A‐c and A‐d). Possible remnants of the fully unfolded meshwork, which may persist after treatment with nocodazole, are indicated by arrows in panels (G‐a) and (G‐b). Largely destructed structures of PSDLs were not noticeable, and the overall structure of the PSDL was largely maintained after treatment with GMP‐PNP, GDP, ATP, and DMSO.

Figure 11 shows the representative images of PSDL structures following exposure to specific reagents, as well as corresponding control samples. Largely destructed structures of PSDLs were notable after the treatment with GTP, nocodazole, and paclitaxel in low magnification EM images (images marked with Lo in Figure 11B,G,H), but were less pronounced or evident after the treatment with GMP‐PNP, GDP, ATP, and DMSO (images marked with Lo in Figure 11C–F).

In the analyses of the remaining PSDL structures with the size of 150 nm to 1.5 μm in high magnification EM images, severely damaged structures were prevalent in the former groups (images marked with S in Figure 11), while structures with nearly normal (N) to less extensively modified (M) were prevalent in the latter groups. Typical examples of normal and severely damaged PSDL structures are shown in each treatment (Figure 11). The interior structures of PSDLs treated with GMP‐PNP and GDP appeared to be unchanged or minimally modified as compared to those of control PSDLs; those incubated with ATP appeared to be more sparse than those incubated with GMP‐PNP and GDP. However, the difference in the effects between these two groups appears to be relatively small, considering the effect of GTP.

The effects of incubation with DMSO, a solvent for nocodazole and paclitaxel, were determined to be destructive based on observations made at low magnification (Figure 11F‐a). The damage observed after treatment with nocodazole and paclitaxel (Figure 11G,H) dissolved in 0.1% DMSO appeared to be more severe than that observed after treatment with DMSO. These results suggest that, in addition to the damage caused by DMSO, treatment with nocodazole and paclitaxel caused further damage.

Globular structures were observed in PSDLs after incubation irrespective of the presence or absence of reagents. Examples of such structures are indicated by the open arrowheads in Figure 11A. Some PSDLs exhibited multiple small globular structures (diameter < 60 nm), while others exhibited a single large globular structure (diameter: 80–110 nm) or did not exhibit any such structures. Such structures were not observed in samples before incubation with the GTB. Therefore, this structure may have been produced or become apparent after incubation with GTB at 0°C. These structures were not further analyzed in this study.

### 3.8 Effects of MT‐Affective Reagents on Non‐Aggregated PSD Structures

To evaluate the effects of the reagents on PSDs, we used two types of PSDs, OG12 and TX‐PSD, as polymerizing MTs interacted with both aggregated and non‐aggregated PSDs (Figures 6, 7, 8). The effects of MT‐affective reagents on non‐aggregated PSDs were evaluated experimentally using OG12 in the same way as the assays for the PSDLs.

**FIGURE 12:**
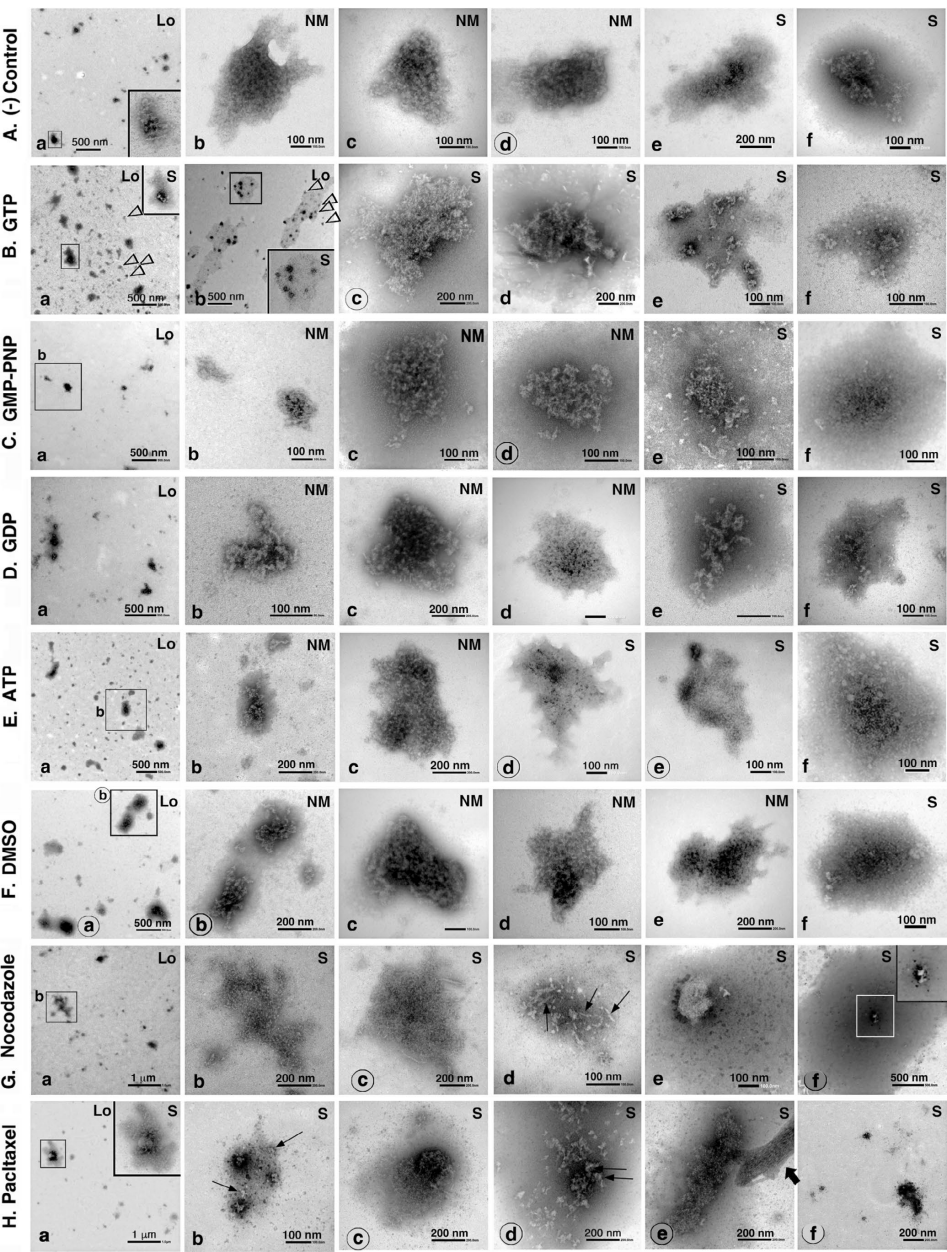
Effects of MT‐affective reagents on non‐aggregated PSDs (OG12). OG12 was treated, and the effects of the treatments on the PSD structures were assessed in the same manner as described in the legend of Figure 11. Morphology of the PSDs after the reagent treatment was categorized into severely damaged (S), and normal to nearly normal (N). Representative images are shown. Largely destructed structures of PSDs with many small dark spots, as typically shown in (B‐a,b) (open arrowheads indicate some of them) were not observed or less frequent after treatment with GMP‐PNP, GDP, ATP, and DMSO. The boxed portions of the structures observed at low magnification are enlarged in the same images or the adjacent right‐hand images. The enlarged structures of (A‐a), (C‐b), (E‐b), and (F‐b) show normal appearances, whereas those of (B‐a) and (H‐a) are damaged. Arrows in (G‐d) and (H‐b and H‐d) indicate thin fiber structures and small aggregated structures that remained in the severely damaged structures following nocodazole and paclitaxel, respectively (not all of such structures are indicated.). The large closed arrows in (H‐e) indicate newly formed structures and possibly bundled MTs following treatment with paclitaxel.

Figure 12 shows representative images of non‐aggregated PSD structures following exposure to the specific reagents and the corresponding control treatments. Following incubation with 10 mM GTP, the number of small fragments was observed to increase (open arrowheads in Figure 12B‐a,b) at low magnification observation, and most of the dark areas do not include normally maintained PSD structures as confirmed at higher magnifications (Figure 12B‐a insert). The results suggest large‐scale destruction of PSD structures after the incubation with GTP. Such large‐scale destructions were also observed after treatments with nocodazole and paclitaxel. The destruction of non‐aggregated PSDs was also observed following incubation with 1 mM GTP (Figure S5F). On the contrary, large‐scale destructions were either not observed or observed less frequently in low‐magnification EMs after treatments with GMP‐PNP, GDP, ATP, and DMSO (Figure 12C‐F). These results suggest that non‐aggregated PSD structures were significantly destroyed following incubation with 10 mM GTP, nocodazole, and paclitaxel.

The destruction of the PSD structures was confirmed at higher magnifications, as shown in Figure 12. Through the observation of the remaining PSDs in high magnification EM images, the morphologies of the PSDs were classified into two groups: normal to moderately damaged (N) and severely damaged (S). It was not easy to classify the structures into three groups as was done in PSDLs. The structures judged to be severely damaged were relatively abundant even in the control sample, which results in the presence of an abundance of them in the structures treated with GNP‐PNP, GDP, ATP, and DMSO. However, treatment with GTP, nocodazole, and paclitaxel resulted in a higher occupancy of severely damaged PSDs and greater structural damage than treatment with GNP‐PNP, GDP, ATP, or DMSO: the former destroyed overall morphology, whereas the latter did so rarely. The globular structures observed in PSDLs (Figure 12) were not observed in the non‐aggregated PSDs.

After treatment with nocodazole and paclitaxel, observation at both low and high magnifications revealed damage to non‐aggregated PSD structures (Figure 12G,H). The nocodazole treatment tends to leave fine fibrous structures (arrows in Figure 12G‐d), while the paclitaxel treatment tends to leave small aggregated structures (arrows in Figure 12H‐b,d). These results may be due to the difference in the actions of the two reagents. A new structure, possibly aggregated tubulin or bundled MTs, appeared after treatment with paclitaxel (thick arrow in Figure 12H‐e), providing evidence of the activity of paclitaxel. After incubation with DMSO, changes in the morphology of non‐aggregated PSDs were less severe at higher magnification compared to the damage caused by nocodazole and paclitaxel (Figure 12F).

The nocodazole treatment tends to leave fine fibrous structures (arrows in Figure 12G‐d), while the paclitaxel treatment tends to leave small aggregated structures (arrows in Figure 12H‐b,d). These results may be due to the difference in the actions of the two reagents. A new structure, possibly aggregated tubulin or bundled MTs, appeared after treatment with paclitaxel (thick arrow in Figure 12H‐e), providing evidence of the activity of paclitaxel. After incubation with DMSO, changes in the morphology of non‐aggregated PSDs were less severe at higher magnification compared to the damage caused by nocodazole and paclitaxel (Figure 12F).

Following treatment with GMP‐PNP and GDP, no increase in the number of small fragments was observed at low magnification. In contrast, after treatment with ATP, an increase in the number of small fragments was observed at low magnification, and PSD structures filled with fine materials were seen at higher magnifications (Figure 12E). However, the damage observed after treatment with GMP‐PNP, GDP, and ATP was less severe compared to the damage caused by GTP, nocodazole, and paclitaxel.

In summary, the damage caused by GTP, nocodazole, and paclitaxel was more severe than that caused by GMP‐PNP, GDP, ATP, and DMSO.

### 3.9 Effects of MT‐Affective Reagents on Aggregated PSD Structures

**FIGURE 13:**
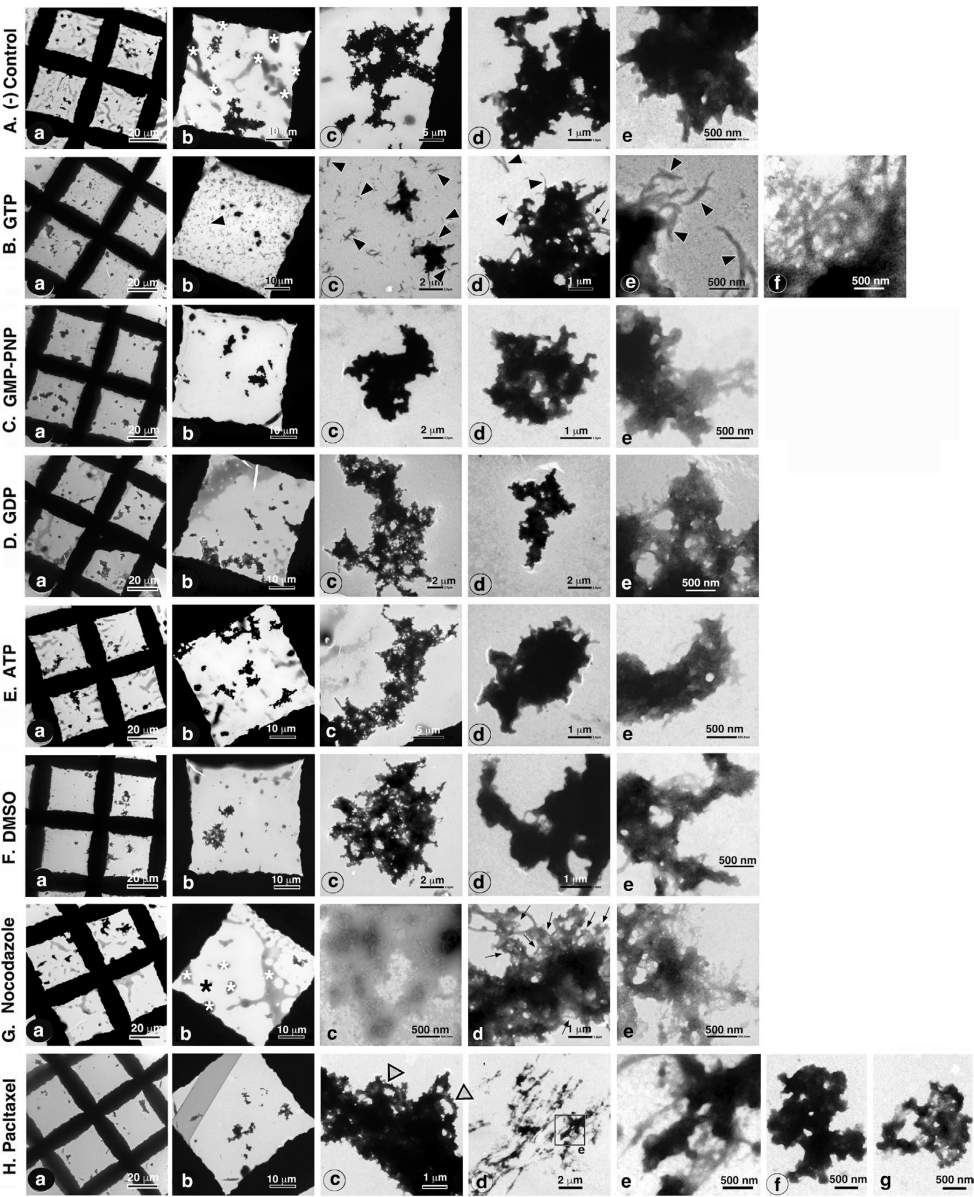
Effects of MT‐affective reagents on PSD aggregates. TX‐PSD was treated in the same manner as described in the legend for Figure 11. Representative images at different magnifications are presented. Differences in the size of the aggregated PSDs across various treatments are most clearly seen in images taken at 600× magnification, which are displayed in the second column from the left. The rightmost images taken at 15 000× magnification, reveal internal structures, which can be observed in the less electron‐dense rims of the aggregates or in areas that appear disorganized due to certain treatments. Some widely distributed short fibers and small bundles of short fibers are indicated by arrowheads in (B). Loosening of structures is indicated by arrows (B‐d and G‐d). Some PSD aggregates became highly electron‐dense, while others were skinny following paclitaxel treatment (H‐c and H‐d, respectively). Heavy decoration of the aggregates, particularly those observed as thickened fibers, is indicated by open triangles in (H‐c). Possible PSD aggregate remnants consisting of a single or a few PSDs are shown in (H‐f) and (H‐g). Image (H‐e) is an enlargement of a boxed area in (H‐d). Areas slightly lighter than PSD aggregates, indicated by white asterisks in (A‐b and G‐b), are nano‐W deposits.

The effects of MT‐affective reagents on aggregated PSDs were evaluated using TX‐PSD. Figure 13 presents representative images of PSD structures after exposure to specific reagents, along with corresponding control samples. The assessment of aggregated PSDs primarily reposed on the reduction of large aggregates. Individual PSDs could not be identified due to their embedding in highly electron‐dense aggregates, with few exceptions. As a result, the assessment protocol used for PSDLs and non‐aggregated PSDs is not applicable. Instead, changes in the size of the PSD aggregates serve as a reliable markers for detecting the deleterious effects of the reagents, alongside other signs of deterioration (This assessment protocol cannot be practically applied to the PSDL and non‐aggregated PSDs.).

**FIGURE 14:**
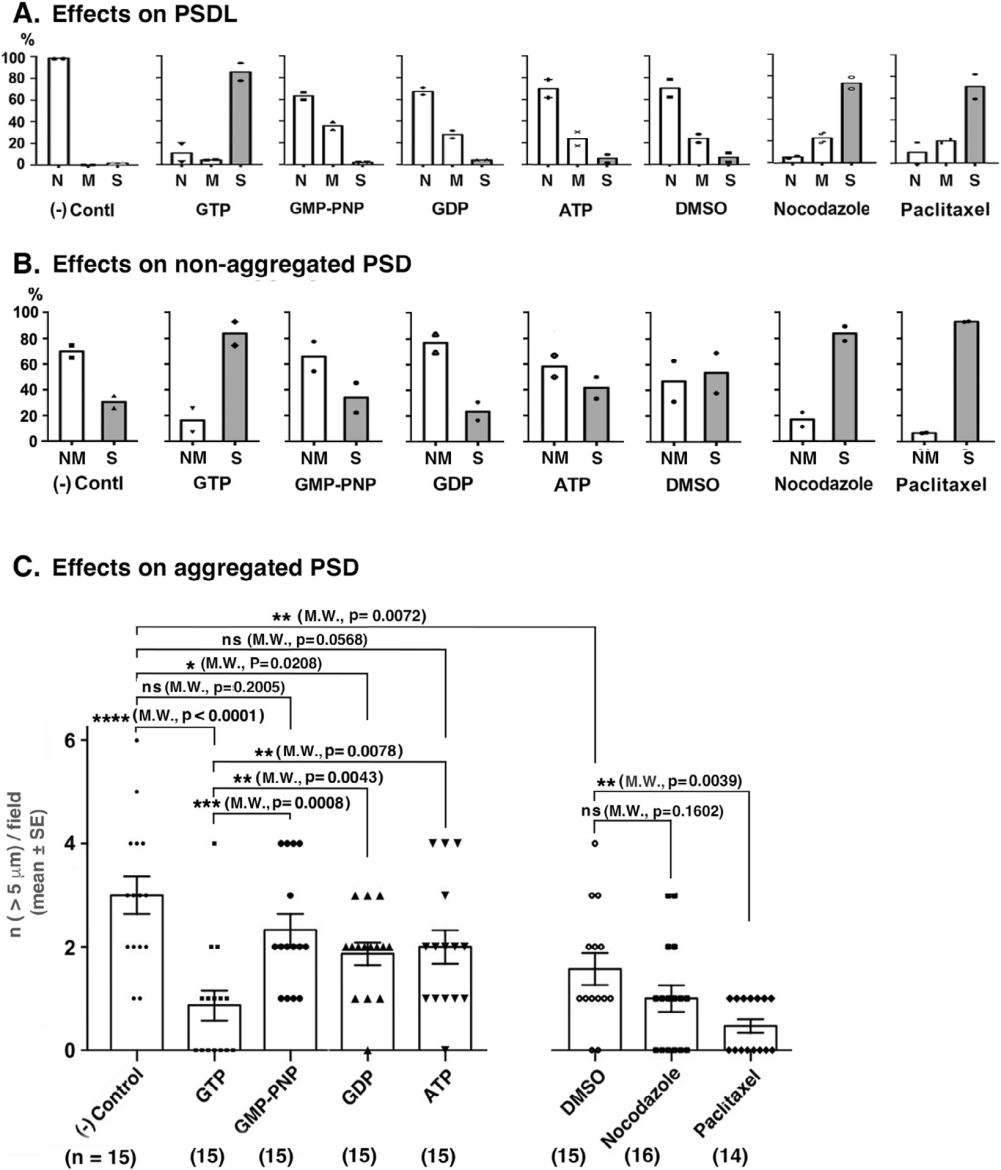
Quantification of the effects of MT‐affective reagents on the PSDL, the non‐aggregated, and aggregated PSDs. The effects of MT‐affective reagents on the structures of the PSDL (A), non‐aggregated PSDs (B), and aggregated PSDs (C) were examined. Samples were treated, and the effects on their structures were assessed as described in the figure legends of Figures 11, 12, 13. (A) The morphology of PSDL structures following various treatments was classified into three categories: normal to nearly normal (N), moderately damaged (M), and severely damaged (S). The abundance of N, M, and S morphologies in each sample is shown, with the percentages plotted. Bars represent the average values (*n* = 2, with 47 or more PSDLs counted per sample). The PSD structures, following various treatments, were classified into two groups: normal to moderately damaged (NM) and severely damaged (S). The abundance of NM and S morphologies in each sample is shown, with percentages plotted. Bars represent the average values (*n* = 2, with 42 or more PSDs counted per sample). (C) EM images were captured randomly at 200× magnification for quantification, and particle sizes and numbers within a single square field of the EM grid were determined through particle analysis using the ImageJ software. The number of remaining large PSD aggregates, defined as those with areas greater than 19.625 μm^2^ (equivalent to the area of a circle with a diameter of 5 μm), is shown. The number of fields counted ranged from 14 to 16 per sample, as indicated at the bottom. Statistical significance was assessed using the Mann–Whitney *U* test. *Statistical significance. *p*‐values are presented in parentheses. ns: not significant.

EM images from a single grid visual field following each treatment illustrate the characteristic size distribution of PSD aggregates following each treatment (leftmost images in Figure 13). Quantification of the number of large aggregated PSDs of which the area is larger than 19.625 μm^2^ (equivalent to the area of a circle with a diameter of 5 μm) in one visual field is shown in Figure 14C. Statistical analyses indicate a significant reduction of the large PSD aggregates after treatment with GTP, despite insignificant changes after treatments with GMP‐PNP, GDP, and ATP. Statistical analyses between GTP‐treated samples and those treated with GMP‐PNP, GDP, and ATP confirmed the absence or weakness of the destructive effects by the latter groups compared with GTP. Treatments with DMSO, nocodazole, and paclitaxel also significantly reduced the large aggregated PSDs. Reductions after the treatment with paclitaxel but not nocodazole were significantly greater than those after the treatment with DMSO, a solvent for paclitaxel and nocodazole. Nocodazole treatment showed a tendency for greater reduction than that after DMSO treatment, but it was not significant.

Another criterion for assessing the destructive effects of the reagents on aggregated PSDs is the presence of significant signs of destruction in the PSD aggregates: the appearance of characteristic short fibers after treatment with 10 mM GTP (Figure 13B‐e), the presence of the area in which PSD aggregates disappeared after treatment with nocodazole (13G‐b and 13G‐c), and the presence of an extremely sparse structure after the treatment with paclitaxel (13H‐d). Such extremely sparse structures were observed after the treatment with 10 mM GTP for 5 days (Figure S6). These signs were not observed after the treatments with GMP‐PNP, GDP, ATP, and DMSO. The third point helpful for the evaluation of deterioration effects is severe loosening (Figure 13B‐f,G‐e).

The way of destruction after the GTP, nocodazole, and paclitaxel treatments appears to differ from the morphological changes in each treatment (see Figure 13B,G,H). Drastic changes in PSD aggregation were observed following incubation with 10 mM GTP. There was a decrease in the size of PSD aggregates, as stated above, and many short fibers, either single or bundled, were widely distributed (arrowheads in Figure 13B). The average diameter of the individual fibers was approximately 20.8 ± 0.7 nm, indicating MT derivatives. As concerns large PSD aggregates, which were relatively few after the treatment, loosening of structures was observed, and short fibers were associated with PSD aggregates (Figure 13B‐d,e). After incubation with GTP, these short fibers were observed in PSD aggregates, but not in non‐aggregated PSDs. This finding suggests that large PSD aggregates unfolded through the peeling off of thin fibers, which may have been the bundling elements of the PSD aggregates. This robust destruction of PSD aggregates, particularly the shedding of short fibers, was not observed in PSD aggregates following treatments with the other reagents. The destruction of PSD aggregates was also observed following incubation with 1 mM GTP (Figure S5H).

Individual PSDs, possibly released from the aggregates, were also severely damaged (Figure 13G‐c). Fiber bundles, possibly twining around PSDs before nocodazole treatment, were loosened in the remaining PSD aggregates (Figure 13G‐d), suggesting the destruction of the aggregates. The fiber structures associated with the remaining PSD aggregates following nocodazole treatment were different from those observed following GTP treatment. Paclitaxel treatment also appeared to cause widespread severe damage to some aggregated PSDs (Figure 13H). The change following paclitaxel treatment is unique in that the possibly destructed PSD aggregated structures (open arrowheads in Figure 13H‐c) were associated with many fiber bundles. In addition, paclitaxel treatment induced the thickening and increased electron density of component fibers during the process leading to the destruction (Figure 13H‐c,f).

In summary, these results suggest that GTP, nocodazole, and paclitaxel exert destructive effects on aggregated PSDs, while changes in PSD aggregates were less severe following treatment with GMP‐PNP, GDP, ATP, and DMSO.

Quantitative analyses on the effects of the above reagents on PSDL, non‐aggregated PSD, are collectively presented in Figure 14. The occupancy of the severely damaged PSDLs and non‐aggregated PSDs after the treatments is extremely high after treatment with GTP, nocodazole, and paclitaxel compared to the other treatments. This is also the case for the aggregated PSDs. Note that the degree of destruction may be much greater than what is displayed by the graph after treatment with GTP, nocodazole, and paclitaxel, as severely damaged structures that fragmented into small, widely dispersed pieces were excluded from being counted.

### 3.10 Fiber Structures in the PSD Aggregates

**FIGURE 15:**
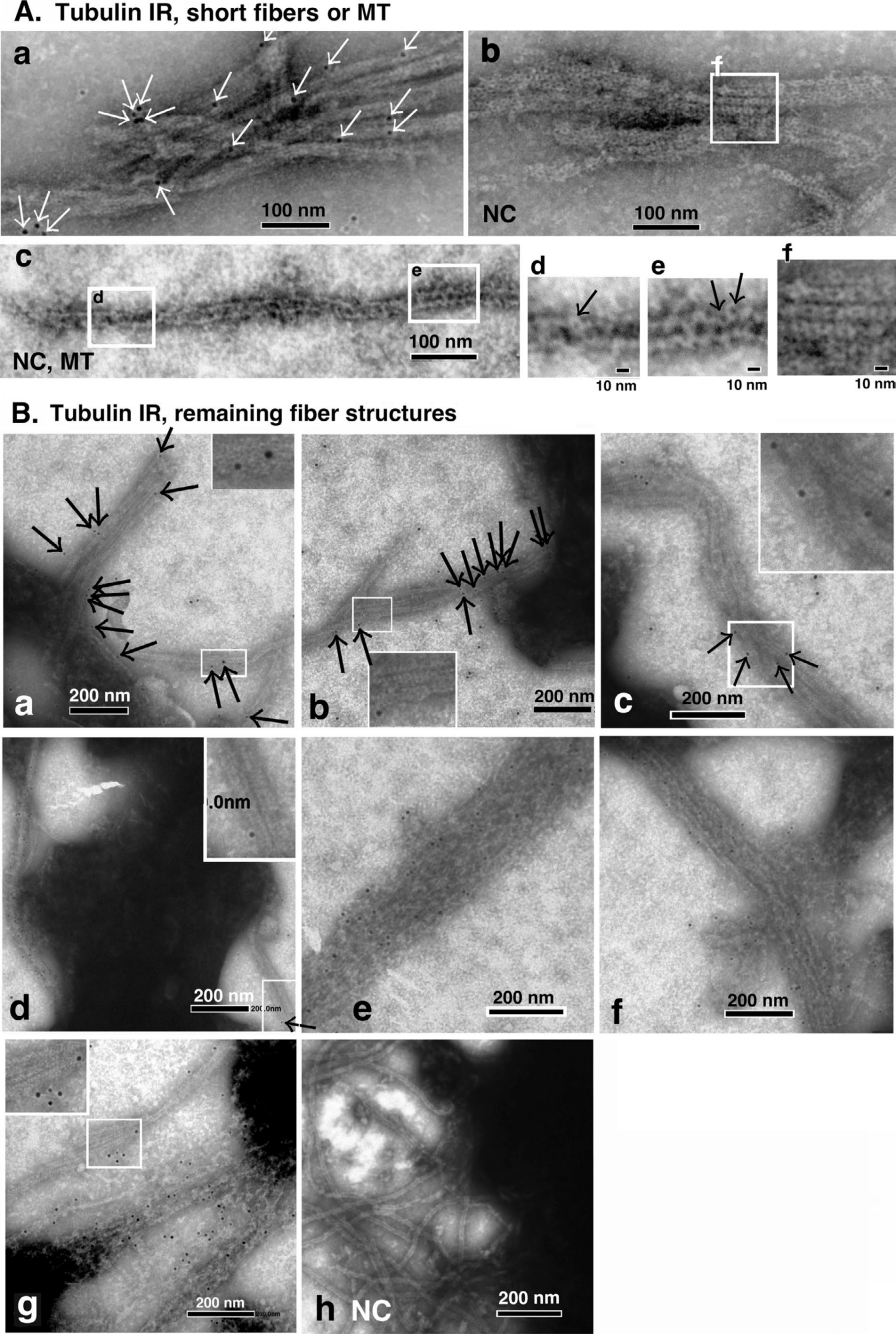
Identification of tubulin through immuno‐gold negative staining EM in GTP‐treated TX‐PSD. TX‐PSD treated with 10 mM GTP at 0°C for 90 min was transferred to a Formvar membrane on the EM grid, fixed with 0.25% glutaraldehyde at 0°C for 5 min, labeled with anti‐tubulin polyclonal antibodies, followed by gold (10 nm)‐labeled secondary antibodies, and negatively stained. Fixation immediately after treatment with GTP was necessary to prevent the loss of short fibers produced following treatment with GTP. Blockade of the specimens was omitted as this was found to be unnecessary. (A‐a) Tubulin immunoreactivity (IR) on the short fibers produced following the treatment of TX‐PSD with GTP. Tubulin‐immunoreactive gold particles are indicated with white arrows. (A‐b), (A‐c) Negative control (NC) prepared by treating TX‐PSD with 10 mM GTP or MTs derived from ML116 tubulin, polymerized through incubation at 37°C for 5 min. Specimens were treated as described in (A‐a), but without the use of primary antibodies (see Figure S4C,D for positive and negative control, respectively, consisting of MTs generated from ML116). Enlarged images of the boxed areas in (A‐b) and (A‐c) are shown in (A‐d), (A‐e), and (A‐f). The fine structures of short fibers produced through treatment with GTP typically consisted of characteristic bundled bead‐like structures, which were different from those of normal MT fibers. Black arrows in (A‐d) and (A‐e) indicate incomplete bead‐like structures in normal MT fibers, suggesting commonality between the two fibers, that is, short fibers were derived from MTs or formed in a similar way as MTs. (B) Tubulin IR on the fiber structures remaining in TX‐PSD aggregates following treatment with 10 mM GTP. Tubulin‐immunoreactive gold particles are indicated by black arrows in (B‐a), (B‐b), (B‐c), and (B‐d), except for those in the inserts. Immuno‐gold particles in (B‐e, B‐f, and B‐g) are not indicated because their distribution was locally abundant. The enlarged views of the boxed portions are shown in the inserts to depict the bead‐like structures seen in (A‐b). (B‐h) Negative control for the immuno‐gold detection experiment, in which primary antibodies were omitted, showing the absence of immuno‐gold particles (see also Figure S4 for immuno‐gold labeled tubulin in the TX‐PSDs during and after interaction with polymerizing MTs).The detailed structures of the short fibers, as well as those of the remaining fibers associated with PSD aggregates, are shown in Figure 15A‐b,f. The fibers were bundled into three or four strands consisting of connected ring structures with a typical diameter of 12.6 nm each (Figure 15A‐f). The detailed structures of negatively stained normal MTs (Figure 15A‐c) were different from those of short fibers. Similar but imperfect ring‐like structures were occasionally observed in normal MTs (arrows in Figure 15A‐d,e), and were not regularly aligned in a line. The presence of imperfect ring‐like structures in normal MTs suggested a similarity between the short fibers and normal MTs. Thus, the short fibers may have been derived from MTs or formed in a similar manner as MTs. However, the molecular organization of the short fibers appeared to be slightly different or changed from that of normal MTs.

To clarify the involvement of tubulin and MTs in PSD aggregation, the presence of tubulin molecules on short fibers and related fibrous structures remaining in the PSD aggregates following treatment with GTP was investigated through immuno‐gold negative staining EM. Tubulin immunoreactivity was detected in short fibers (Figure 15A‐a). This detection was judged to be specific, as negative controls not treated with primary antibodies showed no immuno‐gold particles (Figure 15A‐b) in these fibers and in normal MTs produced in vitro at 37°C (Figure S4D). The labeling was not homogenously distributed in both normal MTs and the short fibers.

The detailed structures of the short fibers, as well as those of the remaining fibers associated with PSD aggregates, are shown in Figure 15A‐b,f. The fibers were bundled into three or four strands consisting of connected ring structures with a typical diameter of 12.6 nm each (Figure 15A‐b). The detailed structures of negatively stained normal MTs (Figure 15A‐c) were different from those of short fibers. Similar but imperfect ring‐like structures were occasionally observed in normal MTs (arrows in Figure 15A‐d,e), and were not regularly aligned in a line. The presence of imperfect ring‐like structures in normal MTs suggested a similarity between short fibers and normal MTs. Thus, the short fibers may have been derived from MTs or formed in a similar manner as MTs. However, the molecular organization of the short fibers appeared to be slightly different or changed from that of normal MTs.

These bead‐like structures were also identified in the remaining fibrous structures associated with PSD aggregates following GTP treatment, and some of them were enlarged (Figure 15B‐a,b,c,d,g). In addition, these bead‐like structures sometimes appeared like ladders depending on the viewing conditions (e.g., Figure 15B‐b,c). As of now, these bead‐like structures have not been observed in the TX‐PSD without 10 mM GTP treatment, although their visibility might have depended on the staining conditions. Therefore, the bead‐like structures may be specific to the type of PSD aggregates we are dealing with.

Tubulin immuno‐gold particles were also widely distributed on other types of fiber bundles associated with PSD aggregates (Figure 15B‐e–g). A similar tubulin immunoreactivity distribution was observed in the fiber structures of TX‐PSDs incubated with polymerizing MTs (Figure S4B). These findings suggested that all these fiber bundles were related to tubulin/MTs; in addition, they suggested that short fibers, as well as those associated with PSD aggregates produced after GTP treatment, may be related to MTs, and that treatment with GTP may induce the release of short fibers from PSD aggregates. Fiber bundles not consisting of bead‐like structures may also be derived from MTs.

Finally, the bead‐like structures were not observed in the TX‐PSD following treatment with nocodazole (Figure S7), in which TX‐PSD structures were disorganized and loosened, as shown in Figure 13G‐d. The results suggest the difference in the mechanism for TX‐PSD aggregate destruction between GTP and nocodazole treatments.

## 4 Discussion

### 4.1 Tubulin Is an Essential Building Block for the PSD Structure

This study revealed that treatment with GTP and MT‐affective reagents, such as nocodazole and paclitaxel, led to the degradation of both PSDLs and non‐aggregated PSDs. This study is the first to report on agents that directly cause PSD deterioration, other than broad‐spectrum proteases and urea (Blomberg et al. 1977; Jan et al. 2002). These findings strongly imply tubulin's fundamental role in the formation and maintenance of PSD.

We also found that PSDL‐like structures are present in purified tubulin preparations (both MAP‐rich tubulin/ML116 and > 99% pure tubulin/T240 preparations). In addition to the overall similarity in morphology, despite variations in specific details (Figure 3; Figure S2), associability with polymerizing MTs was also similar to that of PSDLs (Figure 3F). A structure similar to the PSDL has been identified during purified tubulin polymerization through cryo‐EM (Chrétien et al. 1995). However, such PSDL‐like structures have not been further investigated. While the meshwork structures found in pure tubulin preparations and PSDLs are not identical, they may share certain properties. T240 tubulin has been shown to self‐assemble into a large structure that is distinct from PSDL‐like structures (Samson et al. 2012). This finding is noteworthy, as it suggests the potential of tubulin to form structures other than the well‐characterized MTs. The finding supports the idea that, depending on the conditions, PSDL‐like meshwork structures or bona fide PSDLs can appear in highly purified tubulin or in the brain postsynaptic area, respectively.

The effect of MT‐affective agents on PSDs and the importance of tubulin or MTs suggested by our in vitro study agree with the studies carried out under more physiological conditions. Nocodazole treatment of hippocampal slices reduced the number of dendritic spines, concomitantly increased filopodia, and suppressed long‐term potentiation induced by high‐frequency stimulation. These changes are caused by an interference in the maturation of the dendritic spine via MTs intruding into spines from the dendritic shaft (Jaworski et al. 2009). A similar effect was confirmed in hippocampal neuronal cultures (Jaworski et al. 2009; Penazzi et al. 2016). In addition, another study indicates that colchicine stereotaxically injected into the rat hippocampus reduces the expression of tubulin in the PSD, which is related to synaptic loss (Wu et al. 2020). Similar spine loss was also observed in cultures of the hippocampus from APP transgenic mice, and epothilone, an MT‐stabilizing agent, reverses this type of spine loss (Penazzi et al. 2016). Thus, accumulating publications suggest that events involving PSD‐tubulin also play an important role in synaptic function in vivo.

Mechanistically, the destructive effects of GTP on the structural changes in PSDL and PSD appear to be GTP‐specific and require GTP hydrolysis based on the weakened activities with GTP analogues and an unhydrolyzable analog, GMP‐PNP (Figures 11, 12, and 14). This property suggests the involvement of molecules with GTPase activity, such as heterotrimeric G proteins, small G proteins, dynamin, tubulin, initiation factors, and elongation factors. Not a few of these molecules are present in the purified PSDL used in this study (see Tables S3 and S4 in (Suzuki et al. 2021)). Among these molecules, tubulin is likely the primary contributor to the phenomenon, as it is the most abundant component of PSDL and the disruption of PSDL and PSD is induced by nocodazole and paclitaxel, although other molecules cannot be entirely ruled out. GTP‐induced PSD disassembly may likely occur in vivo, as PSDL and non‐aggregated PSD degradation were observed following treatment with 1 mM GTP (Figure S5), which is close to the physiological concentration of GTP in cells (Sumita et al. 2016; Traut 1994). It is also possible that the deteriorating effect of GTP on the PSDL and PSD is part of the system to reorganize PSD because GTP is one type of second messenger, a regulator of various kinds of cell function. Further studies are needed.

### 4.2 A Possible Linkage Between PSD‐MT Interaction and Synaptic Plasticity

In this study, the interaction of PSDL and non‐aggregated PSD structures with polymerizing MTs was identified through EM observations. This interaction appears to be specific, as indicated by the characteristic changes in the structures after the interaction between PSDL and MTs (Figures 4 and 5), as well as the binding of tubulin to the purified PSDs shown in co‐sedimentation analysis (Figure 10A) and the distinct binding characteristics compared to latex beads (Figure 10B–D). Our data imply that MTs have the capacity to associate with PSDs during the expression of synaptic plasticity as they transiently intrude into the spine head, as suggested before based on the experiments carried out using live image confocal microscopy or total internal reflectance microscopy (Gu et al. 2008; Hu et al. 2011; Hu et al. 2008; Jaworski et al. 2009; Merriam et al. 2013; Schätzle et al. 2018). Our findings on the interaction of PSDLs and non‐aggregated PSDs with polymerizing MTs were corroborated by EM examinations conducted on brain sections (Gray et al. 1982; Westrum and Gray 1976; Westrum and Gray 1977; Westrum et al. 1980), because the morphological features of MTs associating with PSD are similar to those observed in our study. Therefore, these data suggest that the interaction between PSDLs/PSDs and polymerizing MTs occurs in vivo.

The end region morphology of MTs connected to PSDLs/PSDs, including dilatation, distortion, and tapering of the MT cylindrical structure (Figure 5; Figure S3), was similar to those observed in the polymerization of purified tubulin (Akhmanova and Steinmetz 2008; Gudimchuk and McIntosh 2021). Thus, the interaction between PSDLs/PSDs and MT may be similar to that observed when tubulin dimers are incorporated into elongating MT ends. This suggests that the interaction between PSDLs/PSDs and MTs occurs via their tubulin moieties.

PSDLs/PSDs may not function as nucleation sites from where MTs extend as purified PSDLs do not possess γ‐tubulin (Suzuki et al. 2021), which is an essential component for MT nucleation (Lüders and Stearns 2007; Sanchez and Feldman 2017). Tubulin may start polymerization spontaneously with a sufficient concentration of tubulin, elongate, and contact PSDLs/PSDs. When one MT end gets in contact with PSDLs/PSDs, elongation occurs at the other end, and the MT reaches and connects with another PSDL/PSD. The binding of PSDLs/PSDs to both MT ends and interactions occurring through the non‐end MT region and multiple associations between MTs and PSDLs/PSDs allow the enlargement of the PSD area.

The interaction between polymerizing MTs and PSDs seemed to induce the formation or growth of PSDs or PSD‐like structures as suggested in Figure 6B. This alteration in PSD characteristics may be accompanied by a restructuring of the PSD morphology, given the diverse range of morphologies observed in non‐aggregated PSDs (Figures 6C and 9A,B). These diverse PSD morphologies include segmentation, segregation, perforation, and a horseshoe shape, all of which are deemed essential for inducing multiple innervation and increasing synaptic input into a single spine during the development of synaptic plasticity (Sun et al. 2021). Furthermore, the protrusions and interconnections observed in non‐aggregated PSDs (Figure 9A,B) could potentially be remnants of interactions with polymerizing MTs, as well as tubulin‐positive fiber bundles present in the PSD aggregates (Figure 9C; Figure S4). The heterogeneity observed in isolated non‐aggregated PSDs not subjected to in vitro incubation with polymerizing MTs suggests that a similar interaction with polymerizing MTs occurs in vivo. These changes in PSDs may help in the expression of synaptic plasticity, together with increased material supply from outside the spine head to the PSD region through a new connection between the PSD and invading MTs (Hu et al. 2008).

This study also observed changes possibly occurring on the MT side (Figure 5E,G) following the mixing of PSDLs and tubulin and the induction of MT formation, suggesting an influence from PSDLs on MTs. Thus, the effects may be mutual, which could be favorable for the expression of synaptic plasticity. Although further experiments have not been conducted, the mechanism underlying this phenomenon should be explored in future studies.

In summary, tubulin in the PSD may be involved in triggering synaptic plasticity at activated synapses. The absence of effects on the PSDLs after the interaction with polymerizing MTs and subsequent depolymerization may be due to a lack of certain protein(s) within PSDLs.

### 4.3 Tubulin and MTs Are Also Involved in PSD Aggregation and Disaggregation

While examining PSDs through EM, we encountered instances of aggregated PSDs. This occurrence was due to the fact that isolated PSD samples inherently contain a certain degree of aggregated PSDs. It has been shown that conventional PSDs easily aggregate after purification, particularly after freezing in the absence of glycerol at a high concentration (Cohen et al. 1977; Suzuki et al. 2019). We expanded our research focus to include PSD aggregation because our study suggested that postsynaptic tubulin or MT is a key molecule in its formation and rearrangement.

Aggregated PSDs interacted with MTs upon incubation with tubulin under polymerization conditions (Figures 7 and 8). MTs were connected to aggregated PSDs at selected positions on the aggregates, and the number of connected MTs increased, leading to the consequent enlargement of the PSD aggregates (Figure 7). These enlarged PSD aggregates persisted even after MT depolymerization at 0°C, indicating the establishment of stable structures. PSD aggregates present in the TX‐PSD sample underwent substantial breakdown upon exposure to GTP, being transformed into smaller structures and shedding many tubulin‐containing short filaments, potentially originating from fragmented unbundled PSD aggregates (Figures 13 and 15). This deteriorating effect appeared to be related to MT assembly/disassembly because, among the nucleotides examined, the effect was specific to GTP and required GTP hydrolysis (Figure 14C) (Wade 2009), and nocodazole and paclitaxel also deconstructed the aggregates (Figures 13 and 14). This study suggests that PSD aggregation is induced by the intermingling of PSDs with polymerizing MTs. In addition, there was a change in the morphological properties of MTs, particularly MT bundles, as evidenced by the protrusions and fiber bundles in Figure 9C, shown in more detail in Figure 15. Thus, the MT bundles became stabilized upon interaction with aggregated PSDs, whereas unchanged MTs located far outside of aggregated PSDs were depolymerized at 0^o^C. Apparently, the interacting PSDs are stabilized and increase in size because of this mechanism. Such MT‐mediated interactions were predominantly manifested within aggregated PSDs. If an identical or analogous mechanism is potentially applicable to non‐aggregated PSDs, even to a small degree, PSDs could undergo the morphological alterations necessary for synaptic plasticity.

The accumulation of tubulin in PSDs before the homogenization of the dissected and minced brain has been reported (Carlin et al. 1982). Thus, an abnormal association occurs between tubulin or MTs and PSDs under ex vivo conditions. To our knowledge, however, there are no reports on PSD aggregation as observed in this study occurring in vivo. Abnormal aggregation of PSDs might also occur in vivo, but it might not be easy to find. Alternately, large‐scale PSD aggregation like those observed under in vitro conditions is not likely to occur because a single or small number of PSDs, in particular excitatory ones, are usually compartmentalized in dendritic spines. Therefore, the intermingling of many PSDs with massive polymerizing MTs is not likely to occur under in vivo conditions, as far as spine membrane is intact. Instead, small‐scale enlargement of a single or a few to several PSDs might be possible. Under certain pathological conditions, PSDs may aggregate in vivo. Given that PSD aggregation implies a loss of its normal function, exploring such brain pathologies could be valuable.

### 4.4 Effect of GTP on the PSD Integrity

**FIGURE 16:**
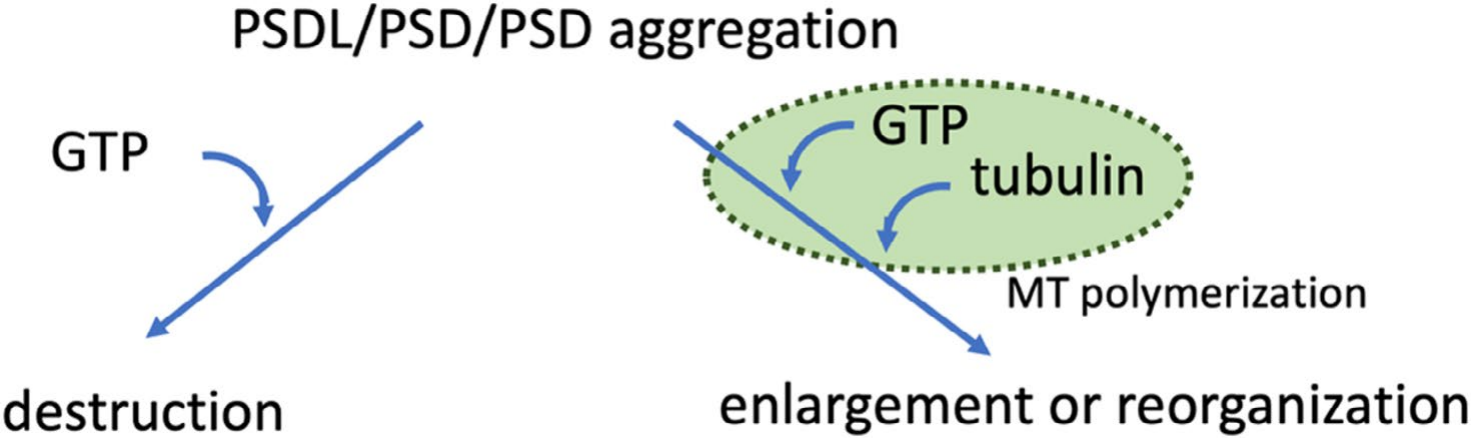
Summary of the GTP effects on the PSDL/PSD/PSD aggregation. Results of the experiments carried out in this study (interaction of postsynaptic structures with polymerizing tubulin, and effects of MT‐affective reagents on postsynaptic structures) are integrated. Postsynaptic structures were incubated with GTP either in the presence or absence of polymerizing tubulin, resulting differentially in the structures' destruction and enlargement/reorganization, respectively. Thus, GTP appears to be a key molecule for PSD structure maintenance and change.

We integrate findings from two types of in vitro experiments conducted in this study: analyses of the interaction between PSDL/PSD and polymerizing tubulin, and the effects of GTP on the structure of PSDL/PSD. These experimental systems were identical except for the presence or absence of polymerizing tubulin and the temperature. Notably, the results of these experiments showed the opposite effects, as shown in Figure 16. The integrated results suggest that the PSD structure is inherently dynamic, balancing between destruction and construction, and this balance can be influenced by the presence of growing MTs. All necessary elements—GTP, soluble tubulin, and MTs—may be present or can be supplied in the postsynaptic sub‐compartment, the dendritic spine, although the changes in GTP concentration within the spine are currently unknown. Therefore, the scheme illustrated in Figure 16 may also be relevant to the in vivo postsynaptic region. Future experiments conducted under in vivo conditions are anticipated.

Tubulin is a pivotal molecular component that influences both the physiological and pathological facets of PSD. Given that the MT cytoskeleton, like the actin cytoskeleton, is fundamental to synaptic function and that dysregulation of this cytoskeleton is critical in many neuronal diseases (Pena‐Ortega et al. 2022; Verstraelen et al. 2017), further investigation into the relationship between PSD and tubulin or MTs is highly required.

## Author Contributions

**Tatsuo Suzuki:** conceptualization, investigation, funding acquisition, data curation, formal analysis, project administration, methodology, validation, visualization, writing – review and editing, writing – original draft. **Toshihiro Fujii:** resources, validation, methodology. **Kiyokazu Kametani:** methodology, investigation. **Weidong Li:** funding acquisition, validation. **Katsuhiko Tabuchi:** funding acquisition, validation.

## Conflicts of Interest

The authors declare no conflicts of interest.

### Peer Review

The peer review history for this article is available at https://www.webofscience.com/api/gateway/wos/peer‐review/10.1111/jnc.70085.

## Supporting information


Data S1.

## Data Availability

The data that support the findings of this study are available on request from the corresponding author. The data are not publicly available due to privacy or ethical restrictions.
